# Genome-Wide Identification and Expression Analysis of *SS* and *SE* Gene Families in *Platycodon grandiflorum*

**DOI:** 10.3390/biology15080620

**Published:** 2026-04-16

**Authors:** Meitong Pan, Junbai Ma, Denghua Wen, Lingyang Kong, Shan Jiang, Panpan Wang, Xiaozhuang Zhang, Weichao Ren, Wei Ma, Xiubo Liu

**Affiliations:** 1 College of Pharmacy, Heilongjiang University of Chinese Medicine, Harbin 150040, China; 2 College of Pharmacy, Heilongjiang University of Chinese Medicine, Jiamusi 154007, China

**Keywords:** *SS* gene family, *SE* gene family, *Platycodon grandiflorum*, triterpenoid saponins, expression pattern

## Abstract

At present, the characteristics of key enzyme genes in the upstream pathway for triterpenoid saponin biosynthesis in *P. grandiflorum*, as well as their expression patterns over the growth duration, have not been systematically analyzed. This study, at the whole-genome level, conducts the first bioinformatics and expression analyses of the *SS* and *SE* gene families in *P. grandiflorum*. Four *PgSS* and seven *PgSE* genes were identified and distributed across six chromosomes. Members within the same subfamily exhibited highly conserved sequences and structures, while distinct structural divergence was observed between different subfamilies. Phylogenetic analysis showed that *PgSS* and *PgSE* genes were closely related to those of dicotyledons such as *Panax ginseng* and *Polygala tenuifolia*, suggesting high evolutionary conservation. Promoter analysis revealed abundant light- and hormone-responsive elements and MYB/MYC binding sites, indicating regulation by multiple signals. Protein secondary structures were dominated by the Alpha helix and were structurally stable. Quantitative real-time polymerase chain reaction (qPCR) demonstrated that expression levels of *PgSS* and *PgSE* in one-year-old Platycodonis Radix were significantly higher than in perennial Platycodonis Radix, especially for the *PgSE* family. This study characterized the basic biological features and growth-stage-dependent expression patterns of the *SS* and *SE* gene families in *P. grandiflorum*. The results identify key candidate genes and molecular targets for regulating triterpenoid saponin biosynthesis, and provide data supporting quality improvement and active metabolite research in this medicinal plant.

## Simple Summary

This study identified and analyzed the *SS* and *SE* gene families of *P. grandiflorum*. Their sequences and evolution are conserved, their structures are stable, and they are regulated by multiple signals. Gene expression in annual plants is more significant than in perennial plants, which provides support for research on saponins mechanisms and variety improvement.

## 1. Introduction

*P. grandiflorum* is a perennial herb of the genus Platycodon in the Campanulaceae family [1]. Dried Platycodonis Radix are traditional and classic medicinal parts [2]. It is widely distributed across China, South Korea, Mongolia, Japan, and Russia [3]. It was first documented in Shennong’s *Classic of Materia Medica* over 2000 years ago. It has remarkable therapeutic effects in the clinical treatment of respiratory diseases and can effectively alleviate cough symptoms induced by low-temperature stimulation and heat-related pathogenic factors [4,5]. It also has multiple effects, including alleviating sore throat and swelling, promoting the discharge of lung secretions, enhancing the targeted delivery of drugs to the respiratory tract, and regulating the body’s immune function. Currently, it is often used to treat sore throat and swelling, purulent lung infections with expectoration of pus, recurrent respiratory tract infections, and acute lung injury [6]. Modern pharmacological research has confirmed that platycodin is the characteristic active component of the plant *P. grandiflorum* and the core substance through which *P. grandiflorum* exerts its pharmacological effects. It belongs to the triterpenoid saponin class of compounds, with a complex, diverse structure that includes multiple monomer components, such as Platycodin D and Platycodin C [7]. A large number of studies have shown that platycodin has multiple biological activities, including anti-inflammation, antioxidation, immune regulation, cardiovascular protection, anti-fibrosis, and neuroprotection [8]. In terms of anti-inflammatory effects, platycodin can reduce various inflammatory responses by inhibiting the release of inflammatory factors such as IL-1β and TNF-α [9]. In antioxidant research, total saponins of *P. grandiflorum* and individual saponins [7] such as Platycodin D can inhibit the generation of reactive oxygen species (ROS) and malondialdehyde (MDA), and increase the levels of antioxidant substances such as glutathione (GSH), superoxide dismutase (SOD), and glutathione peroxidase (GSH-Px), thereby resisting the damage caused by oxidative stress [7]. Platycodin, with its diverse biological activities, has significant potential in fields such as drug research and functional food development.

However, there are still many deficiencies in our current understanding of the biosynthetic pathway of platycodin, which, to a certain extent, greatly restricts the in-depth development and utilization of *P. grandiflorum*’s medicinal value. The biosynthesis of plants is a complex metabolic process involving multiple enzymatic reaction steps and the coordinated regulation of numerous genes. Generally speaking, the synthesis of triterpenoid saponins begins in the mevalonate (MVA) pathway in the cytoplasm and the 2-C-methyl-D-erythritol-4-phosphate (MEP) pathway in the plastids [9]. These two pathways lead to the synthesis of isopentenyl diphosphate (IPP) and its isomer dimethylallyl diphosphate (DMAPP) [10]. Subsequently, IPP and DMAPP are polymerized in sequence to form farnesyl diphosphate (FPP). FPP is converted into squalene under the action of squalene synthase (SS) [11,12]. Squalene is further oxidized by squalene epoxidase (SE) to for m 2,3-oxidized squalene. 2,3-oxidized squalene serves as a key precursor substance [13]. Under the action of a series of oxidases and glycosyltransferase enzymes, it undergoes multiple reactions to ultimately synthesize various structurally complex triterpenoid saponins [14]. SS and SE, as key enzymes in the biosynthesis pathway of triterpenoid saponins, play a crucial role in connecting the previous and subsequent stages of the entire metabolic process [15]. SS catalyzes the condensation of two FPP molecules to form squalene, an important branching point in the synthesis pathway of triterpenoid saponins [16]. It determines the allocation of metabolic flow from FPP to triterpene synthesis, while SE converts squalene into 2,3-oxidized squalene [16]. The enzymes also provide direct precursors for the subsequent cyclization and modification reactions of triterpenoid saponins [14]. Therefore, an in-depth study of the functions of the *SS* and *SE* gene families in *P. grandiflorum* is crucial for comprehensively analyzing the biosynthetic pathway of platycodin and revealing the molecular mechanism underlying the formation of the medicinal active components of *P. grandiflorum*. In recent years, significant progress has been made in genomic research on *P. grandiflorum*. However, a complete genome identification and expression analysis of the *SS* and *SE* gene families of *P. grandiflorum* have not yet been revealed. There is still a lack of a systematic, in-depth understanding of the identification of family members, gene structure, phylogenetic relationships, promoter cis-regulatory elements, and expression patterns across different tissues and developmental stages of these family members. This study aims to characterize the *SS* and *SE* gene families in *P. grandiflorum* at the whole-genome level, including their basic features and functional properties. Using qPCR, we compared the expression patterns of these gene families in Platycodonis Radix between one-year-old and perennial *P. grandiflorum*, and analyzed the relationship between target gene expression and growth year. Our results provide a theoretical foundation and valuable genetic resources for future studies on variety improvement and the biosynthetic regulation of triterpenoid saponins in *P. grandiflorum*.

## 2. Materials and Methods

### 2.1. Materials

This experiment takes *P. grandiflorum* as the research object. On 9 October 2022, mature seeds of *P. grandiflorum* were collected from the medicinal plant garden of Heilongjiang University of Chinese Medicine. Seeds with plump grains, uniform size, intact seed coats, and no damage from pests or diseases were selected to ensure consistent germination. The seedling substrate was prepared by mixing garden soil and vermiculite in a 3:1 volume ratio. The substrate was sterilized by high-pressure steam for 30 min to remove pathogenic microorganisms and weed seeds. We sowed the screened seeds in the seedling pots, with 3 seeds in each pot and a sowing depth of 1 cm. We then filled the seedling pots with sterilized mixed substrate. All seedling pots were placed in the Medicinal Plant Garden of Heilongjiang University of Chinese Medicine for cultivation at 23 °C and 80% light intensity, and in neutral soil (pH ≈ 7.0). We irrigated once every five days. After the seeds germinated, we performed thinning. We kept one healthy seedling in each pot and continued cultivating under the above stable conditions. After the plants had grown for the required years, random sampling was conducted on the healthy and uniformly growing *P. grandiflorum* samples using the random sampling method. Experimental materials for annual (harvested in October 2023) and perennial (harvested in October 2025) *P. grandiflorum* plants were obtained. All statistical analyses presented in this study included both technical and biological replicates. Platycodins Radix tissues were collected. Fresh samples were immediately stored in liquid nitrogen after collection and used for subsequent experiments. Genomic data of *P. grandiflorum* is available from NCBI database (https://www.ncbi.nlm.nih.gov/nuccore/JACONP000000000, accessed on 14 March 2024) [17]. The sequence information of SS and SE proteins of *A. thaliana* was obtained from the TAIR database (https://www.arabidopsis.org/, accessed on 16 March 2025).

### 2.2. Identification, Physicochemical Property Analysis, and Subcellular Localization Prediction of Members of the P. grandiflorum SS and SE Gene Families

Using protein sequences from the SS and SE families of *A. thaliana* as reference sequences, Blast alignment analysis was performed on the genome data of *P. grandiflorum* using TBtools software (V.2.441) (E-value ≤ 1 × 10^−5^, with other parameters set to default values of TBtools). Hidden Markov models corresponding to SS proteins (PF00494) and SE proteins (PF08491) were downloaded from the InterPro database (https://www.ebi.ac.uk/interpro/search/sequence/, accessed on 18 March 2025). The Simple HMM Search function in TBtools was used to search the full protein sequence of *P. grandiflorum* (using TBtools’ default parameters) to obtain HMM-filtered candidate sequences. The two sets of candidate sequences were merged, and the intersection was selected as the credible members of the *SS* and *SE* in *P. grandiflorum*. These sequences were uploaded to the NCBI Conserved Domains Database (https://www.ncbi.nlm.nih.gov/, accessed on 20 March 2025) to verify the integrity of SS and SE conserved domains, ensuring the accuracy and reliability of the identification results. The key physicochemical parameters of PgSS and PgSE proteins, including amino acid residue length, molecular weight (MW), and theoretical isoelectric point (pI), were predicted and analyzed using Expasy (https://web.expasy.org/compute_pi/, accessed on 22 March 2025). The subcellular localization prediction of the PgSS and PgSE was conducted using the CELLO v.2.5 online tool (http://cello.life.nctu.edu.tw/, accessed on 26 March 2025).

### 2.3. The Chromosomal Positions of PgSS and PgSE Genes and the Analysis of Intraspecific Collinearity

Using the gff3 annotation file for the *P. grandiflorum* genome and the predicted gene IDs, the position information of *SS* and *SE* gene family members was extracted. The positions of the genes on the chromosomes were visualized using the Gene Location Visualization tool from GTF/GFF and the Gene Density Profile function in TBtools [17]. By combining the entire genome sequence of *P. grandiflorum* obtained from the public database with the GFF annotation file, the TBtools software was used to filter and select the protein-coding genes with clear chromosome localization, eliminate the unanchored chromosome gene sequences, conduct gene sequence alignment and clustering of collinear blocks within the entire genome, and construct an interspecies collinearity analysis model [18]. Using the chromosome of *P. grandiflorum* as the reference ring, the positions of the *PgSS* and *PgSE* genes were marked. Homologous genes were linked by colored lines. Different colors represented different collinear blocks. The chromosome length was scaled proportionally. The chromosome number and length (unit: Mb) were labeled. The sequence similarity of collinear homologous genes was verified through BLAST 2.17.0 comparison to ensure the reliability of the collinearity relationship. Quantitative analysis was conducted to determine the degree of collinearity between different genomes of the same species.

### 2.4. Analysis of the Phylogenetic Tree of the SS and SE Gene Families in Six Different Plant Species

In total, 40 SS protein sequences and 51 SE protein sequences of 5 representative plants were obtained from the public database, including *Arabidopsis thaliana*, *Oryza sativa*, *Polygala tenuifolia*, *Eleutherococcus senticosus*, and *Panax ginseng*. *A. thaliana* is a classic model plant with well-annotated gene functions, serving as a reference for homologous gene alignment. *O. sativa* is a monocotyledon used to distinguish evolutionary differences between monocotyledons and dicotyledons. *P. ginseng* is a medicinal plant with a well-characterized triterpenoid saponin biosynthesis pathway, providing core references for homologous functional annotation. *P. tenuifolia* is a traditional medicinal plant rich in triterpenoid saponins, with similar metabolic characteristics to *P. grandiflorum*. *E. senticosus* is a medicinal plant belonging to Araliaceae, and it is phylogenetically closely related to *P. grandiflorum* as a dicotyledon. The protein sequence files of *A. thaliana* were downloaded from the TAIR database, and the protein sequence files of *O. sativa* were downloaded from the Rice Genome Annotation Project Database (RGAP) [19]. We downloaded the *P. tenuifolia* protein sequence file from the Beijing Institute of Genomics (BIG) database [20] and the protein sequence file for *E. senticosus* from the China National GeneBank Database (CNGBdb) [19]. We obtained the *P. ginseng* protein sequence file from the GigaDB database (accessed on 8 May 2025) [21]. The protein sequences of six plant species are listed in Tables S2 and S3. The phylogenetic tree was constructed using the Neighbor-Joining (NJ) method with the JTT (Jones–Taylor–Thornton) amino acid substitution model in MEGA software (V.11.0.13), based on the sequence alignment. The obtained *P. grandiflorum* SS and SE sequences were compared with the protein sequences of *A. thaliana*, *O. sativa*, *P. tenuifolia*, *E. senticosus*, and *P. ginseng*, respectively, using the MUSCLE function of MEGA software for multiple sequence alignment. Using the PHYLOGENY function of MEGA11 software, the Bootstrap value was set to 1000 repetitions, and the Newick Tree file was exported [22]. Then, the visualization and beautification of the phylogenetic tree was completed through the ITOL online tool (https://itol.embl.de/personal_page.cgi, accessed on 18 May 2025).

### 2.5. The Gene Structure of P. grandiflorum SS and SE: Analysis of Conserved Motifs, Domains, Introns and Exons

Using the TBtools software, the introns and exons of the *PgSS* and *PgSE* genes were identified and extracted from the *P. grandiflorum* genomic structure annotation file. The MEME online analysis tool was used to identify the conserved motifs of the *P. grandiflorum* SS and SE proteins. The number of motifs was set to 10, and other parameters were set according to the system defaults. The generated mast.xml format file was downloaded. Through the Batch CD-Search function in the NCBI database (https://www.ncbi.nlm.nih.gov/Structure/bwrpsb/bwrpsb.cgi, accessed on 24 May 2025), the conserved domains of the PgSSs and PgSEs were downloaded. Then, combined with the phylogenetic tree of PgSSs and PgSEs and the gene structure annotation file GFF, the joint analysis and visualization drawing of gene structure, conserved motifs, domains, and intron–exon structure were conducted using the TBtools software.

### 2.6. Analysis of Cis-Acting Elements in the Promoter Regions of PgSS and PgSE Genes and Prediction of the Secondary Structures of PgSS and PgSE Proteins

Based on the GFF structure annotation file of the *P. grandiflorum* genome and the full-length sequences of the *PgSS* and *PgSE* genes, using the Promoter Extraction function of the TBtools software, the 2000 bp sequence upstream of the promoters was extracted. The filtered *PgSS* and *PgSE* promoter sequences were submitted to the online analysis tool PlantCARE (https://bioinformatics.psb.ugent.be/webtools/plantcare/html/, accessed on 14 June 2025) using default parameters to predict the composition of cis-regulatory elements (CREs) in these promoter regions. Based on the predicted CRE counts, a visual analysis was conducted using the HeatMap function in TBtools. The secondary structure of the PgSS and PgSE protein sequences was predicted using the SOPMA online tool (https://npsa-prabi.ibcp.fr/cgi-bin/npsa_automat.pl?page=/NPSA/npsa_sopma.html, accessed on 14 June 2025), resulting in four conformations: α-helix, extended chain, β-turn, and random coil [23].

### 2.7. Extraction, Quality Detection and qPCR Verification of Total RNA from P. grandiflorum

Total RNA was extracted from the Radix Platycodin samples of annual and perennial *P. grandiflorum* using the Plant Total RNA Extraction Kit (Simgen Biotechnology Co., Ltd., Hangzhou, China). The extracted RNA was quickly quality-checked by 1% agarose gel electrophoresis, and the A260/A280 and A260/A230 ratios were measured using an ultramicro UV–visible spectrophotometer to determine RNA purity. The extracted RNA was reverse-transcribed into cDNA using the HiScript^®^III RT SuperMix for qPCR (+gDNA wiper) reverse transcription kit (Nanjing Vazyme Biotech Co., Ltd., Nanjing, China), and the concentration of the obtained cDNA was determined using an ultramicro UV–visible spectrophotometer. The primers used in this experiment were designed using Primer3web (V.4.1.1) (https://ginkgo.zju.edu.cn/genome/tools/primer3/, accessed on 27 October 2025), and the primer sequences are shown in Supplementary Table S1. Using the qPCR reagent kit ChamQ Universal SYBR QPCR Master Mix (Nanjing Vazyme Biotech Co., Ltd., Nanjing, China) and a real-time fluorescence quantitative PCR instrument (Bio-Rad Laboratories (Shanghai, China) Co., Ltd.), the expression of the *SS* and *SE* genes was detected by qPCR. The internal reference gene selected was *PgGAPDG*. The PCR reaction procedure was as follows: pre-denaturation at 95 °C for 30 s; 40 amplification cycles, 95 °C for 10 s → 60 °C for 30 s; 95 °C for 30 s → 65 °C for 30 s → 95 °C for 30 s. This generated the melting curve of the sample. Three technical replicates and three independent biological replicates were set for each sample. The results are expressed as average values and variances, and the relative expression levels of *PgSS* and *PgSE* candidate genes were calculated using the2^®−ΔΔCt^ method.

### 2.8. Prediction of Protein Interaction Network Between PgSS and PgSE Proteins

We uploaded the PgSS and PgSE protein sequences to the STRING Version 12.0 database and set the required interaction score to medium confidence (0.400). At the same time, we checked “hide disconnected nodes in the network”. By default, all types of evidence were retained. Subsequent analysis should primarily focus on interpreting the experimental verification and co-expression data. After the PgSS and PgSE protein predictions were completed, we output the TSV file and imported it into Cytoscape 3.10 to beautify the protein interaction network diagram.

## 3. Result

### 3.1. Identification of SS and SE Gene Family Members, Physicochemical Property Analysis and Subcellular Localization Prediction of P. grandiflorum

In this study, the *SS* and *SE* gene family members of *P. grandiflorum* were systematically analyzed using the TBtools tool, combined with downloaded Fasta and Gff files of *P. grandiflorum*. Through bidirectional comparison using the BLAST and HMMER function of TBtools software (V.2.441), a total of four *SS* gene family genes were identified, namely *Pgchr02 19270T*, *Pgchr05 35010T*, *Pgchr07 08670T*, and *Pgchr09 01060T*, alongside seven *SE* gene family genes, namely: *Pgchr01 27850T*, *Pgchr02 17330T*, *Pgchr02 20400T*, *Pgchr02 29230T*, *Pgchr03 28780T*, *Pgchr03 28800T*, and *Pgchr05 08870T*. The protein domain analysis of the above 11 genes was conducted using the NCBI website, and it was found that all four PgSS genes contained the (PF00494) conserved domain, and all seven PgSE genes contained the (PF08491) conserved domain. The physicochemical properties of the 11 protein sequences were analyzed using the EXPASY website, and the results of AA length, MW, pI, and subcellular localization are shown in Table 1. The results showed that the longest PgSS protein, PgSS3, contained 439 amino acid residues, the shortest PgSS protein, PgSS1, contained 303 amino acid residues, and the other two sequences had approximately 400 amino acid residues. The MW of PgSS4 was 43.94 kDa, and the rest of the proteins were within 50 kDa. The pI ranged from 6.14 (PgSS4) to 9.03 (PgSS3), and all proteins were located in the cytoplasm. The longest PgSE protein, PgSE1, contained 602 amino acid residues, the shortest PgSE protein, PgSE3, contained 149 amino acid residues, and the other five sequences had amino acid residue numbers ranging from 213 to 589. The MW of PgSE1 was 66.02 kDa, and the rest of the proteins were between 16.84 and 64.33 kDa. The pI ranged from 5.38 (PgSE2) to 9.37 (PgSE3), and four proteins were located in the cytoplasm, while five proteins were located in the inner membrane.

### 3.2. Analysis of the Chromosomal Locations of PgSS and PgSE Genes and Their Intraspecific Collinearity

**Figure 1:**
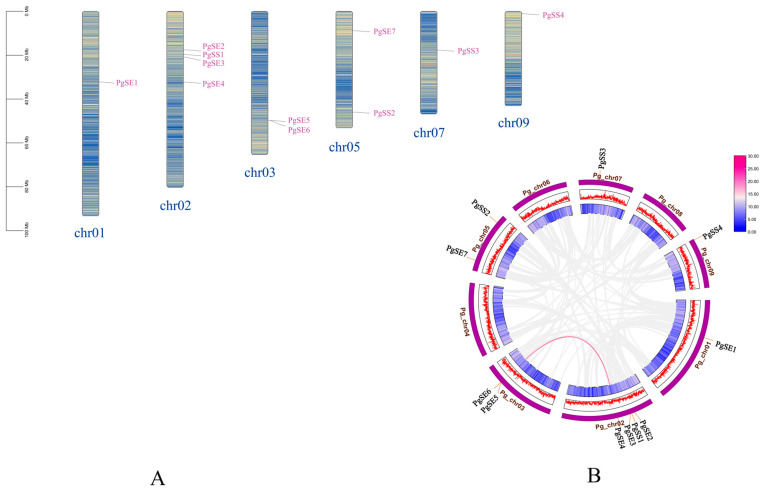
(**A**) The chromosomal distribution of *SS* and *SE* gene family members in *P. grandiflorum*. All identified *PgSS* and *PgSE* genes are mapped to chromosome groups (chr) of *P. grandiflorum*. Each chromosome band is labeled with its corresponding chromosome number at the bottom. A scale of 0–100 Mb indicates the physical length of each chromosome. The red lines on the chromosome indicate a dense distribution of genes. (**B**) Intraspecific collinearity analysis of the *P. grandiflorum SS* and *SE* gene families. Chromosomes are arranged in a circular pattern and distinguished by different-colored blocks. Homologous collinear gene pairs are connected by colored curves. Chromosome numbers and gene IDs are labeled around the periphery to illustrate the intraspecific collinearity relationships.

The 11 genes of the *PgSS* and *PgSE* gene families are distributed on six chromosomes of *P. grandiflorum*, as shown in Figure 1A. According to their distribution on the chromosomes, they are named *PgSS1* to *PgSS4*, and *PgSE1* to *PgSE7*. This study completed data screening and analysis using the TBtools software and established an intraspecific collinearity Circos diagram to systematically reveal the intraspecific collinearity characteristics of *SS* and *SE* genes in *P. grandiflorum*, as shown in Figure 1B. The main conclusions are as follows: in the *SS* family of the *P. grandiflorum*, no collinear gene pairs were found; in the *SE* family of the *P. grandiflorum*, there was one pair of genes, namely *PgSE4* (*Pgchr02 29230T*) and *PgSE5* (*Pgchr03 28780T*); and *PgSE4* and *PgSE5* may have been generated through fragment duplication and replication.

### 3.3. Phylogenetic Tree Analysis of SS and SE Protein Systems in Six Different Plants

**Figure 2:**
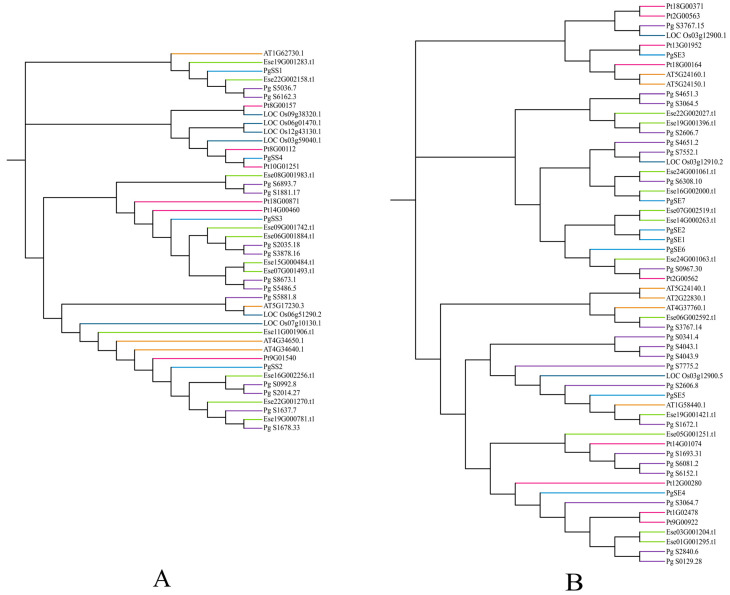
(**A**) Phylogenetic tree of SS protein family from six plants. (**B**) Phylogenetic tree of SE protein family from six plants. Different species are marked with distinct colors, and corresponding gene IDs are labeled alongside.

The phylogenetic tree analysis is shown in Figure 2. Most of the SS and SE proteins form close relationships with dicotyledonous plants such as *P. ginseng* and *P. tenuifolia*. Among them, PgSS4 and Pt10G01251, PgSE3 and Pt13G01952, PgSE7 and Ese16G002000.t1, PgSE1, PgSE2, Ese16G002000.t1 and Ese14G000263.t1 cluster within the same branch. are grouped into the same branch. The SS and SE proteins of *P. grandiflorum* did not form a monophyletic cluster with the SS and SE proteins of *A. thaliana*, while *O. sativa*, as a monocotyledons plant, had SS and SE proteins that formed an independent branch [24,25]. The two families of SS and SE in *P. grandiflorum* both show a phenomenon of partial functional conservation and partial functional differentiation, providing an important basis for further in-depth analysis of the molecular regulatory mechanism of triterpenoid saponin synthesis during the development of *P. grandiflorum* annual and perennial forms [26].

### 3.4. Structure of the SS and SE Genes of P. grandiflorum: Conservation of Motifs, Conservation of Domains, Intron–Exon Analysis

**Figure 3:**
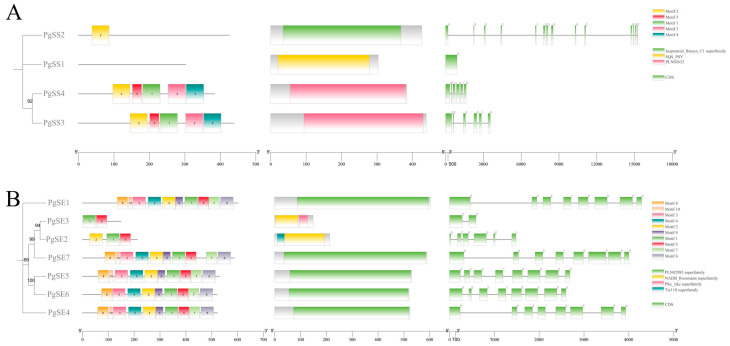
(**A**) The composition and distribution of conserved motifs, analysis of conserved functional domains, and exon–intron structural characterization of PgSSs. (**B**) The composition and distribution of conserved motifs, analysis of conserved functional domains, and exon–intron structural characterization of PgSEs.

From Figure 3A,B, it can be seen that among the four *P. grandiflorum SS* genes, those belonging to the same subfamily, namely *PgSS3* and *PgSS4*, both contain five identical conserved motifs and the PLN02632 conserved domain. *PgSS1* and *PgSS2* have slightly different structures, especially *PgSS1*, which does not show the presence of the conserved motif. Among the seven genes in the *PgSE* family, both contain Motif1 and Motif5. *PgSE1*, *PgSE7*, *PgSE5*, and *PgSE4* contain 10 identical conserved elements. Compared with the *PgSE* motif and PLN02985 domain, the other genes in the *PgSE2* and *PgSE3* subfamily have slightly different structures.

Analyzing the number and distribution characteristics of introns and exons within gene families is of great significance for understanding the evolutionary history, functional differentiation, expression regulation, and molecular mechanisms of gene families [16,27]. Observing the gene structures of members of the *PgSS* gene family and *PgSE* gene family, it was found that among the *PgSS* members, the gene structure of *PgSS1* contains one intron, while *PgSS2* has the largest number of exons (14), and the number of introns and exons for the other members is six each, with similar intron–exon distribution; it was also discovered that in the *PgSE* family, the gene structure of *PgSE3* contains two exons, while *PgSE2* has the largest number of exons (two), and the number of introns and exons for other members is eight each, with similar intron–exon distribution. During the evolutionary process of *P. grandiflorum*, *PgSS1*, *PgSS2*, *PgSE2* and *PgSE3* may have undergone multiple gene splicing events or gene fragment insertion processes, gradually differentiating and leading to the diversification of protein structure and function, possibly through functional specialization to adapt to new environmental conditions and perform protein functions different from the original genes.

### 3.5. Analysis of Cis-Acting Elements in the Promoter Regions of PgSS and PgSE Genes and Prediction of the Secondary Structures of PgSS and PgSE Proteins

**Figure 4:**
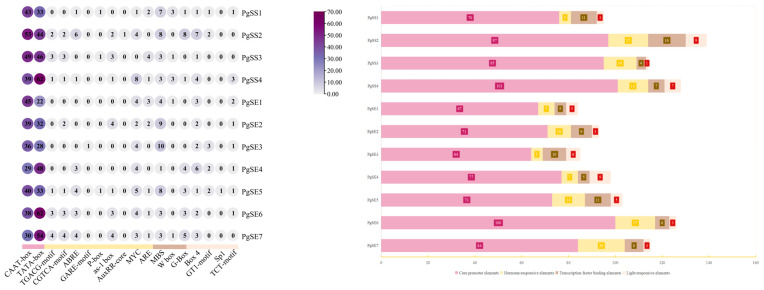
Statistical analysis of cis-acting elements in the promoters of *SS* and *SE* gene family members in *P. grandiflorum*. The cis-acting elements in the promoters were classified into four categories according to their biological functions and marked with different colors.

The analysis of the cis-acting elements in the promoter regions of *PgSS* and *PgSE* genes revealed that approximately 60 cis-acting elements were detected in the promoters of these two genes. All the promoter regions of *PgSS* and *PgSE* contained four types of cis-acting elements: core promoter elements, hormone response elements, transcription factor binding elements, and light response elements. This study selected 18 representative cis-acting elements for statistical mapping. As shown in Figure 4, all members of the *PgSS* and *PgSE* families have abundant CAAT-box and TATA-box core promoter elements, indicating that these two families have a molecular basis for high-level transcription [28]. Additionally, these family members also share MBS transcription factor binding elements and Box 4 light response elements. Among them, *PgSS2* and *PgSE3* have the most MBS binding sites, with 8 and 10, respectively, suggesting that these genes may be regulated by MYB transcription and that fDWnd may participate in abiotic stress responses [29]. *PgSS2*, *PgSE4*, and *PgSE5* have the most Box 4 binding sites in the family, with seven and six, respectively, indicating that these two genes may be significantly regulated by light [30]. In these two families, hormone response elements CGTCA motif/TGACG motif, ABRE, GARE motif, and MYC are widely distributed. *PgSS2*, *PgSS3*, and *PgSE6*, *PgSE7* are enriched in the core elements of the JA signaling pathway, such as the CGTCA and TGACG motifs. *PgSS2* and *PgSE4*, *PgSE5*, *PgSE6*, and *PgSE7* are enriched with ABRE elements, except for *PgSS2* and *PgSE4*, which contain a small amount of ARE elements. It is speculated that this gene is highly regulated by the ABA signal [31]. G-box elements are among the most critical components of the light signal pathway. *PgSS2*, *PgSE4*, *PgSE5*, *PgSE6* and *PgSE7* are enriched for G-box elements, especially in *PgSS2*, which has eight G-box elements. The cis-acting elements in the promoter regions of the *PgSS* and *PgSE* genes provide a crucial basis for elucidating the transcriptional regulatory mechanisms of these two gene families.

The analysis of protein Alpha helix, extended strand, Beta turn, and random coil secondary structures was conducted because secondary structures are more conserved than sequences. Based on structural features, the functions and stabilities of proteins can be inferred, providing important supporting evidence for the classification of the *PgSS* and *PgSE* subfamilies and for their evolutionary relationship [32]. Based on the Sopma website, the secondary structures of the PgSS and PgSE protein families were predicted. The prediction results are shown in Table 2. The results showed that the Alpha helix percentage of PgSS was 48.97–66.67%, the extended strand percentage was 1.65–7.03%, the Beta turn percentage was 0%, and the random coil percentage was 29.81–44.19%; the Alpha helix percentage of PgSE was 36.71–43.40%, the extended strand percentage was 12.21–15.62%, the Beta turn percentage was 0%, and the random coil percentage was 43.40–50.17%. The Alpha helix percentage of the protein sequences of the PgSS and PgSE family members was the highest. Among the members of the PgSS family, PgSS3 and PgSS4, which belong to the same subfamily, have similar secondary structures, while PgSE1 and PgSE4, which belong to independent subfamilies in the PgSE family, have different secondary structures from the other five genes. We speculate that there is a differentiation in the secondary structure composition across subfamilies, and this structural difference may be related to the different functions of the genes.

### 3.6. Expression Analysis (qPCR Expression) of PgSS and PgSE Genes Related to Triterpenoid Saponin Synthesis in P. grandiflorum

**Figure 5:**
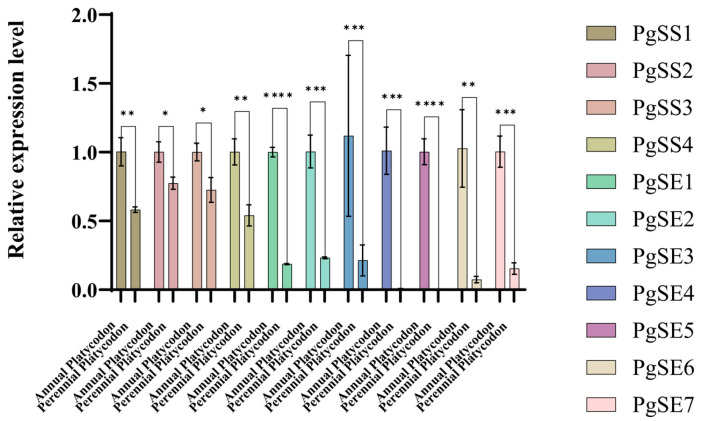
Expression comparison of *SS* and *SE* gene family members in the Radix Platycodins of annual and perennial *P. grandiflorum*. Different genes are shown in distinct colors. Significant differences are indicated as follows: * *p* < 0.05, ** *p* < 0.01, *** *p* < 0.001, **** *p* < 0.0001.

The expression levels of the *PgSS* and *PgSE* family genes in the roots of annual and perennial *P. grandiflorum* were verified by qPCR analysis. As shown in Figure 5, members of the *PgSS* and *PgSE* families generally showed higher expression in annual plants and lower expression in perennial plants. Moreover, the expression level of the *PgSE* family members in annual plants was significantly higher than that in perennial plants, and the difference was extremely significant. According to the figure, both the *PgSS* and *PgSE* families exhibited growth-year-dependent expression patterns in *P. grandiflorum* roots. We speculate that *PgSS* and *PgSE* should be key gene families with specific expression during the development stages of the *P. grandiflorum* Radix Platycodins, and in particular the *PgSE* family may be the core member regulating the response to years and the development stages of the *P. grandiflorum* Radix Platycodins.

### 3.7. Prediction of Protein Interaction Network Between PgSS and PgSE Proteins

**Figure 6:**
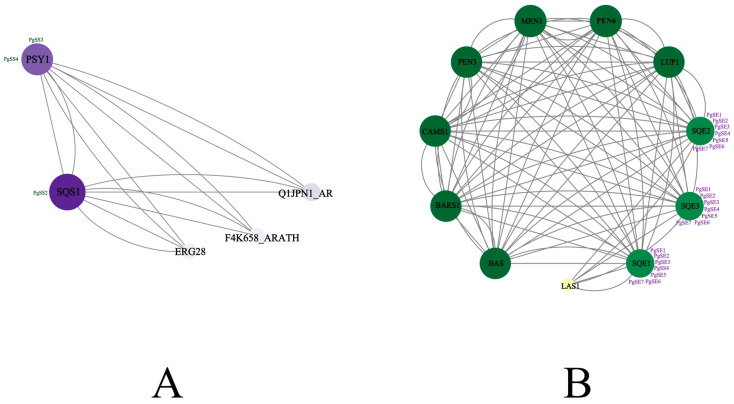
(**A**) PgSS protein network interaction map. (**B**) PgSE protein network interaction map. Lines represent protein interactions, and circles indicate individual protein nodes. The darker the color, the higher the protein’s connectivity and core status within the network.

The network-based analysis of the PgSS and PgSE protein families revealed significant differences in the interaction characteristics of family members. The STING protein interaction analysis of the PgSS family is shown in Figure 6A. In the interaction network, SQS1 shows relatively close connections and may participate in upstream metabolic processes [33]. F23N19.9 is a specific metabolic protein, and SQS2 has no active function. The remaining genes that had no direct association with triterpenoid saponin synthesis were manually excluded. The STING protein interaction analysis of the PgSE family is shown in Figure 6B. After excluding the irrelevant proteins TPS24 and TPS10-2 from the TPS family [34], the genes PEN6, CAMS1, PEN3, LAS1, LUP1, BARS1, MRN1, and BAS formed a highly linked and interconnected network. Among them, the SQE family was the core of the network. The interaction networks of the two families provided key molecular-level evidence for explaining the difference in triterpenoid saponin content between annual and perennial *P. grandiflorum* Radix Platycodins.

## 4. Discussion

*P. grandiflorum*, as a medicinal plant, has roots rich in triterpenoid saponins with anti-inflammatory, liver-protective, anti-tumor, and immune-regulating activities. In the triterpenoid saponin biosynthetic pathway, SS and SE function as key upstream enzymes that participate in regulating metabolism toward triterpenoid saponin synthesis, supply important precursors for downstream reactions, and play important roles in the conversion of carbon sources into secondary metabolites. Through systematic analysis, this study is the first to comprehensively characterize the genomic constitution, chromosomal localization, evolutionary characteristics, and structural divergence of the *SS* and *SE* gene families in *P. grandiflorum*. These findings provide novel insights into the evolutionary patterns of key genes underlying the triterpenoid saponin biosynthetic pathway in this medicinal species [35].

Bioinformatics analysis revealed that all four PgSSs contain the PF00494 conserved domain, while seven PgSEs share the PF08491 conserved domain. Based on subcellular localization predictions and protein secondary structure analysis, the proteins predominantly exhibit α-helical structures with high stability. Considering variations in amino acid length, molecular weight, and isoelectric points among family members, it is inferred that different genes within the same family may perform distinct functions [26]. The analysis of gene structure and conserved motifs indicates that the structures of members of the same subfamily are highly similar, reflecting the characteristic of this family that both conservation and specificity coexist during plant evolution. Moreover, based on the above structural differentiation features, it is speculated that during the long-term evolution process of each subfamily, they may have undergone functional differentiation to adapt to different physiological processes and environmental response patterns, thereby forming structural differences between subfamilies. Subsequently, the expression characteristics and response patterns of the *SS* and *SE* gene family in *P. grandiflorum* can be analyzed through various stress treatment systems [36,37]. For instance, PgSS1 possesses a conserved domain but lacks conserved motifs, yet is clustered with model plants in the phylogenetic tree, indicating structural differences likely resulting from evolutionary sequence divergence. Intraspecific collinearity analysis revealed no collinear gene pairs within the *P. grandiflorum SS* family, whereas only the *SE* family exhibited a single pair of duplicated gene segments. These findings, combined with previous research, suggest that the family as a whole follows a conservative evolutionary trajectory with limited expansion. Current studies on evolutionary patterns of plant *SS* and *SE* gene families remain limited. Future research incorporating whole-genome identification and collinearity analysis of *SS* and *SE* families across more species will facilitate comparative studies of evolutionary characteristics among plants and deeper insights into gene family expansion mechanisms and evolutionary dynamics [38]. Phylogenetic analysis demonstrated that PgSSs and PgSEs cluster closely with dicotyledonous plants such as *P. ginseng* and *P. tenuifolia*, while showing distinct divergence from monocotyledons such as *O. sativa*, further validating the evolutionary conservation of these gene families. Promoter cis-element prediction revealed that *PgSS* and *PgSE* family members exhibit abundant binding sites for light, jasmonic acid (JA), abscisic acid (ABA), and MYB/MYC transcription factors in their promoter regions, suggesting they are more likely to be regulated by such signaling pathways [39,40]. qPCR results showed significantly higher expression levels of *PgSS* and *PgSE* in annual *P. grandiflorum* roots compared with perennial plants, with greater differences observed in *PgSE*. This indicates that annual *P. grandiflorum* is in a vigorous vegetative growth phase with active synthesis of secondary metabolic precursors. The high expression of *PgSS* and *PgSE* provides sufficient precursor materials for triterpenoid saponin synthesis. In contrast, perennial plants shift their metabolic focus toward substance storage [41]. These distinct expression patterns offer novel insights for quality regulation in *P. grandiflorum* cultivation.

Plant research has progressed from a macroscopic perspective to the genetic level. This study preliminarily elucidated the characteristics and expression patterns of the *PgSS* and *PgSE* families through bioinformatics analysis and qPCR, providing precise molecular targets for targeted breeding of high-quality varieties [36]. For instance, research on the medicinal plant *Panax notoginseng* demonstrated that the *PnNAC2* gene positively regulates saponin biosynthesis by binding to the promoters of key biosynthetic genes, including *PnSS*, *PnSE*, and *PnDS*. The saponin synthesis capacity of plants can be predicted by detecting *PnNAC2* binding sites [15]. In *Apocynum venetum*, analysis of *PP2C* gene family members can be used to screen for superior salt-tolerant varieties of *A. venetum* [42]. A whole-transcriptome analysis of the *ARF* gene family in *Polygonatum kingianum*, along with expression profiling of *ARF* members across different tissues, revealed that the *PkARF* gene plays a critical role in root growth, development, and the regulation of secondary metabolism. These findings provide technical solutions for genetic improvement strategies and quality evaluation protocols in *P. kingianum* [43]. At present, our research on *P. grandiflorum* has not involved systematic in vitro enzyme activity analysis or in vivo functional verification on the core genes. In the future, we can select key members with higher expression levels for gene cloning and functional verification, deeply understand the metabolic mechanisms of triterpene saponins, combine gene editing and transcriptional regulation analysis, gradually construct a complete regulatory pathway, and conduct research on the abiotic stress response [38]. This provides a more systematic theoretical basis for molecular mechanism research for the targeted improvement of *P. grandiflorum* quality and the synthesis of triterpenoid saponins.

In summary, this study systematically elucidated the structure, evolution, and expression patterns of the *P. grandiflorum SS* and *SE* gene families, confirming their role as key regulators of the upstream biosynthesis of triterpenoid saponins. This finding provides a novel research paradigm for understanding the molecular regulatory mechanisms underlying the growth cycle, gene expression, and active components of *P. grandiflorum.*

## 5. Conclusions

This study focused on the medicinal plant *P. grandiflorum* and performed a comprehensive genome-wide bioinformatics analysis of the *SS* and *SE* gene families, which are key to the triterpenoid saponin biosynthetic pathway. We comprehensively characterized four *PgSS* and seven *PgSE* genes, including their physicochemical properties, chromosomal localization, intraspecific collinearity, phylogenetic relationships, promoter cis-regulatory elements, and protein secondary structures. Cis-acting element analysis of the promoters suggests that these genes may be regulated by light, hormones, and transcription factors. We therefore speculate that the expression of *PgSSs* and *PgSEs* is closely associated with the developmental stage of *P. grandiflorum*. Protein–protein interaction analysis further uncovered the core regulatory network underlying triterpenoid saponin biosynthesis. These findings provide a novel biological basis and valuable genetic resources for dissecting the biosynthetic mechanism of triterpenoid saponins, as well as for quality improvement and molecular breeding in *P. grandiflorum*.

## Figures and Tables

**Table 1:** Detailed information on genes and proteins of PgSS and PgSE.

Gene Name	Accession Number	Chromosome	AA Length/aa	MW/ku	pI	Predicted Subcellular Localization
*PgSS1*	Pgchr02 19270T	chr2	303	33.78	8.97	Cytoplasmic
*PgSS2*	Pgchr05 35010T	chr5	426	48.68	7.13	Cytoplasmic
*PgSS3*	Pgchr07 08670T	chr7	439	49.52	9.03	Cytoplasmic
*PgSS4*	Pgchr09 01060T	chr9	384	43.94	6.14	Cytoplasmic
*PgSE1*	Pgchr01 27850T	chr1	602	66.02	9.18	Cytoplasmic
*PgSE2*	Pgchr02 17330T	chr2	213	23.50	5.38	Cytoplasmic
*PgSE3*	Pgchr02 20400T	chr2	149	16.84	9.37	Cytoplasmic Inner Membrane
*PgSE4*	Pgchr02 29230T	chr2	523	57.32	8.88	Cytoplasmic Inner Membrane
*PgSE5*	Pgchr03 28780T	chr3	530	57.30	8.99	Inner Membrane
*PgSE6*	Pgchr03 28800T	chr3	520	56.78	7.90	Inner Membrane
*PgSE7*	Pgchr05 08870T	chr5	589	64.33	8.85	Inner Membrane

**Table 2:** Prediction analysis of secondary structure of proteins encoded by PgSS and PgSE families.

Gene Number	Alpha Helix (%)	Extended Strand (%)	Beta Turn (%)	Random Coil (%)
*PgSS1*	63.70	1.65	0.00	34.65
*PgSS2*	66.67	3.52	0.00	29.81
*PgSS3*	48.97	6.83	0.00	44.19
*PgSS4*	53.12	7.03	0.00	39.84
*PgSE1*	36.71	13.12	0.00	50.17
*PgSE2*	41.78	12.21	0.00	46.01
*PgSE3*	41.61	12.75	0.00	45.64
*PgSE4*	43.40	13.19	0.00	43.40
*PgSE5*	40.94	13.58	0.00	45.47
*PgSE6*	42.31	12.69	0.00	45.00
*PgSE7*	37.18	15.62	0.00	47.20

## Data Availability

Raw sequencing data are not publicly available due to institutional data governance restrictions but may be requested from the corresponding author. All experimental data generated or analyzed during this study are available from the corresponding authors upon reasonable request.

## Supplementary Materials

The following supporting information can be downloaded at: https://www.mdpi.com/article/10.3390/biology15080620/s1. Table S1: *PgSS* and *PgSE* primer sequences; Table S2: Protein sequences of *SS* gene families in six different plant species; Table S3: Protein sequences of *SE* gene families in six different plant species.

## Abbreviations

The following abbreviations are used in this manuscript:

ROS	reactive oxygen species
TLAMDA	malondialdehyde
GSH	glutathione
SOD	superoxide dismutase
GSH-Px	glutathione peroxidase
MVA	mevalonate
MEP	the 2-C-methyl-D-erythritol-4-phosphate
IPP	isopentenyl diphosphate
DMAPP	dimethylallyl diphosphate
SS	squalene synthase
SE	squalene epoxidase
AA length	amino acid residue length
MW	molecular weight
pI	theoretical isoelectric point
RGAP	Rice Genome Annotation Project Database
BIG	Beijing Institute of Genomics
CNGBdb	China National GeneBank Database
NJ	neighbor-joining
CREs	cis-regulatory elements
qPCR	quantitative real-time polymerase chain reaction
JTT	Jones–Taylor–Thornton
*P. kingianum*	*Polygonatum kingianum*
*A. venetum*	*Apocynum venetum*
*P. grandiflorum*	*Platycodon grandiflorum*
*A. thaliana*	*Arabidopsis thaliana*
*O. sativa*	*Oryza sativa*
*P. tenuifolia*	*Polygala tenuifolia*
*E. senticosus*	*Eleutherococcus senticosus*
*P. ginseng*	*Panax ginseng*
