# Supplementary material for: Genome-Wide Identification and Expression Analysis of SS and SE Gene Families in Platycodon grandiflorum
Source: Biology (Basel). 2026 Apr 16;15(8):620. doi: 10.3390/biology15080620 (PMC13112935; doi:10.3390/biology15080620)
Supplement: Supplementary file 1 [file biology-15-00620-s001.zip › biology-4240336-supplementary.pdf]

Table S1 *PgSS* and *PgSE* primer sequences

Gene Name	Accession number	（Forward，5'→3'）	（Reverse，5'→3'）
PgSS1	Pgchr0219270T	TGGTGGAAAGAAGCTGTGGA	TTGCGCATCATTGATCCGAG
PgSS2	Pgchr0535010T	GATACCAAAGTCGCGCATGT	GCACAGCCTTGGTTGAGTTT
PgSS3	Pgchr0708670T	TCTGTTGCTCTGATGTGGGT	AAATTCCTCTCCCGGGCTAG
PgSS4	Pgchr0901060T	CTATGTGTGGTGCAGGAGGA	ATCAAGCATGTCGAAAGGGC
PgSE1	Pgchr0127850T	AAGACGAATGTGGCTCCTCA	GATGTGGATCAGCTGGCATG
PgSE2	Pgchr0217330T	TGGAATGACTGTCGCTCTGT	CTTGCGGAGGGTGTAAAAGG
PgSE3	Pgchr0220400T	TGGAATGACTGTCGCTCTGT	TGCCATTAATCTCACCTTGCG
PgSE4	Pgchr0229230T	TCCATCGCCCATCTTGTTCT	GGGATCTGAGGAGCTACGAC
PgSE5	Pgchr0328780T	CTGTTGGGATTTGCTGTGCT	GCAGGCTTTGGGAAACTTCT
PgSE6	Pgchr0328800T	AAGACTGTCGTGGCTTCTCA	ATCCCCAAGTAAGAGTGCCC
PgSE7	Pgchr0508870T	TGGAATGACTGTCGCTCTGT	GCCACTAATCTCACCTTGCG

Table S2 Protein sequences of *SS* gene families in six different plant species

Species	Gene ID	Protein sequence
Eleutherococcus senticosus	Ese07G001493.t1	MSVTLLGVVFPNSEISNVFGYLENAREGSRLLDSSRSIS<br>RDRNLLCTSRLKKGNIHKWSSCSLNPELRYSCLGCTGL<br>KNGGNFPVHSSMVVNPGGEMTVSSEQKVYDVVLKQ<br>AALVKKQLRSSEDLDVKPDIILPGTLSLLSEAYDRCGE<br>VCAEYAKTFYLGTLLMTPERRRAIWAIYVWCRRTDEL<br>VDGPNASHITPSALDRWESRLDDIFKGRPFDMLDAALS<br>DTVTKFPVDIQPFKDMIDGMRMDLKKSRYKNFDELYL<br>YCYYVAGTVGLMSVPIMGIAPDSQATTESVYNAALAL<br>GLANQLTNILRDVGEDARRGRVYLPQDELAQAGLSDE<br>DIFAGKVTDKWRNFMKKQIKRARMFFDEAEKGVTEL<br>SSASRWPVWASLLLYRQILDEIEANDYNNFTRRAYVSK<br>PKKILALPVAYAKALVAPSREASPMVKATRTSNS
	Ese15G000484.t1	MSVALLGVVFPNSEISNVFEYLENAREGSRLLDSSRSIF<br>RDRNLLCTGRHKKDKIHKWSSCSLNAELRYSCLGCSG<br>LENGGKFPVHSSMVVNPAGEITVSSEQKVYDVVLKQA<br>ALVKKHLRSSEDLDVKPDIIFPGTLGLLSEAYDRCGEV<br>CAEYAKTFYLGTLLMTPERRRAIWAIYVWCRRTDELV<br>DGPNASHITPSALDRWESRLDDLFKGCPFDMLDAALS<br>DTVTKFPVDIQPFKDMIDGMRMDLKKSRYKNFDELYL<br>YCYYVAGTVGLMSVPIMGIAPDSLATTESVYNAALAL<br>GIANQLTNILRDVGEDAQRGRVYLPQDELEQAGLSDE<br>DIFAGKVTDKWRNFMKKQIKRARMFFDEAEKGVTEL<br>SSASRWPVWASLLLYRQILDEIEANDYNNFTRRAYVSK<br>PKKILALPVAYAKALVAPSRAASPMVKAP
	Ese08G001983.t1	MAATTFSLVRAVCCPKESNGRFHKKNNGGEITKSRRL<br>RSCSVNNNLSTGGVSALAMASGVVGRSSEERVYEVVL<br>KQAALVREERTTGRPLGFKTTSSSSDYNMSSWDLLNE<br>AYDRCGEVCAEYAKTFYLGTLLMTPERRRAVWAIYV<br>WCRRTDELVDGPNASHITPKALDRWETRLNDLFQGRP<br>YDMYDAALSDAVSTYPVDIQPFKDMIDGMRMDLKKS<br>RYQTFDELYLYCYYVAGTVGLMTVPVMGIAPESKAST<br>ESVYNAALALGIANQLTNILRDVGEDARRGRIYLPQDE<br>LDLAGLTPDYIFRGKVTDKWRRFMKGQIERARMFFNE<br>AEKGVAELSSASRWPVWASLLLYRQILDAIEANDYDN<br>FTKRAYVGKAKKLVSLPVAYGRALLSPSSSVGELLTR
	Ese09G001742.t1	MSVALVWVVSPSSEISNCLGYLEIAREGSRLLDSSRLIS<br>RDRNLNSCRLNKGKIQRWSTGTLNAELRYSCLGSSRL<br>QNGSKFTVHSSMVANPTGEMTLSSEQKVYDVVVKQA<br>ALVKRQLRSSEDLEVKPDIILPGTLSLLSEAYDRCGEVC

Species	Gene ID	Protein sequence
		AEYAKTFYLGTLLMTPERRRAIWAIYVWCRRTDELVD<br>GPNASHITPSALDRWELRLEDLFKGCPFDMLDAALSD<br>TVTKFPVDIQPFKDMIDGMRMDLKKSRYKNFDELYLY<br>CYYVAGTVGLMSVPIMGIALDSQATTESVYNAALALG<br>IANQLTNILRDVGEDARRGRVYLPQDELAQAGLSDEDI<br>FAGKVTDKWRNFMRKQIKRARMFFDKAEKGVTELSP<br>ASRWPVWASLLLYRQILDEIEANDYNNFTKRAYVSKP<br>KKILALPAAYAKSLVAPSRRASPSLKA
	Ese06G001884.t1	MSVALVWVFPTSSEIANCFGYLESVREGSRLLDSSRLIS<br>RDRNLNSDRLKKGKIQRWSSCTLNAELRYSCLGNSRL<br>QNGSKFTVHSSMVANPTGEMTLSSEQKVYDVVLKQA<br>ALVKRQLKSSEDLEVKPDSILPGTLSLLSEAYDRCGEV<br>CAEYAKTFYLGTLLMTLERQRAIWAIYVWCRRTDELV<br>DGPNASHITPSALDRWELRLEDLFKGRPFDMLDAALS<br>DTVSKFPVDIQPFKDMIDGMRMDLKKSRYKNFDELYL<br>YCYYVAGTVGLMSVPIMGIAPDSQAMTESVYNAALA<br>LGIANQLTNILRDVGEDARRGRIYLPQDELAQAGLSDE<br>DIFAGNVSDKWRNFMKKQIKRARMFFDEAEKGVTEL<br>SPASRWPVWASLLLYRQILDEIEANDYNNFTKRAYVSK<br>HKKILALPAAYAKSLVAPSRTASPSLKA
	Ese22G002158.t1	MSGTSASSGLRAAFSYCVQQVRNYDYHHYLCLLELPP<br>DMRRAAFALRAFNVETARAMDVASDPKIGLMRLLWW<br>EEAIDKIFANKLIEHPTAQALACVISEHKVSKRWLKRS<br>VEARINDARREVSDIPQTVNDLEKYAEDTVSTILYTTL<br>QAGGIRSTTADHAASHIGKANGVLLLLKALPYHASRN<br>HHFSYIPAEVAEKHGLLVKQGGQSEIRMDSREGLCDAV<br>FEMASVANAHLEKARELAGKVPAEACPVLLPAVPAQV<br>LLDSLRHVHFDVFDPKLTRGILGSPPLLFQLKLKWHSW<br>REMSEDIEYRCFVGGLSWSTSDRALKEAFEKFGGLVE<br>AKVVVDKESGRSRGFGFVTFDDKHAMDDAIEAMNGI<br>DLDGRNITVDKAQPQGGRDRDSGRGHDRDRGRDRDY<br>GGGGRGGGGRGGGGGECFKCGKPGHFARECPGEGGD<br>RGGRYGGRDDRYGGGRGGGGGRFGPDRNGDRYSGR<br>NRDGGGHGGGDRYNRDRSGPYERR
	Ese16G002256.t1	MGSLGAILKHPDDFYPLLKLKFAARHAEKQIPPEPHW<br>AFCYSMLHKVSRSFGLVIQQLDPQLRDAVCIFYLVLRA<br>LDTVEDDTSIPTEIKVPILMAFHRHIYDKDWHFSCGTK<br>EYKVLMDEFHHVSNAFLELGSGYQEAIEDITMRMGA<br>GMAKFICKEVETINDYDEYCHYVAGLVGLGLSKLFHA<br>SGAEDLATDSLSNSMGLFLQKTNIIRDYLEDINEIPKSR<br>MFWPRQIWSKYVDKLEDLKYEENSAKAVQCLNDMV<br>TNALLHAEDCLKYMSDLRDPAIFRFCAIPQIMAIGTLA<br>LCFNNIQVFRGVVKMRRGLTAKVIDRTKTMSDVYGAF<br>FDFSCLLKSKAQFVIGLSGMSRQPNNGHLIVYVTSKCT<br>EDIAITLPIL
	Ese19G000781.t1	MGSLGAILKHPDDFYPLLKLKIAARHAEKQIPPEPHWA<br>FCYSMLHKVSRSFGLVIQQLGPQLRDAVCIFYLVLRAL<br>DTVEDDTSISTEVKVPILMAFHRHIYDNDWHFSCGTKE<br>YKVLMDEFHHVSNAFLELGSGYKRAIEDITMRMGAG<br>MAKFICKEVETIDDYDEYCHYVAGLVGLGLSKLFHAS<br>GAEDLATDSLSNSMGLFLQKTNIIRDYLEDINEIPKSRM<br>FWPRQIWSKYVDKLEDFKYEENSGKAVQCLNDMVTN<br>ALLHVEDCLKYMSDLRDPAIFRFCAIPQIMAIGTLALC<br>YNNIQVFRGVVKMRRGLTAKVIDRTNTMSDVYGAFF<br>DFSCMLKSKVDKKDPNATKTLSRVEAIQKTCKDSGAL<br>TTKRSNISRVCNRKSYIIENESGYNSTLVSVMLPFDL

Species	Gene ID	Protein sequence
	Ese22G001270.t1	MGSLGAILKHPDDFYPLLKLKIAARHAEKQIPPEPHWAFCYSMLHKVSRSFGLVIQQLGPQLRDAVCIFYLVLRALDTVEDDTSIATEVKVPILIAFHRHIYDNDWHFSCGTKEYKVLMDEFHHVSNAFLELGSGYKEAIEDITMRMGAGMAKFICKEVETIDDYDEYCHYVAGLVGLGLSKLFHASGFEDLATDSVSNSMGLFLQKTNIIRDYLEDINEIPKSRMFWPRQIWSKYADKLEDLKYEENSGKAVQCLNDMVTNTLLHVEDCLKYMSDLRDPAIFRFCAIPQIMAIGTLALCYNNIQVFRGVVKMRRGLTAKVIDRTNTMSDVYGAFFDFSCMLKSKVDNNDPNATKTLSRLEAIQKTCKNSGALTTKRKSYIIENESGYNSTLIVILFIILAILYAYLSSS
	Ese11G001906.t1	MGSMGEILKHPDELYPLVKLIWSVWDAEKQIRREPHWSFCYSMLAKISRSFCLVIHQLSPQLRDALCIFYLVLRALDTVEDDMSIPTEVKEPILMAFHRHIYDTDWHFSCGEKEYKILMDEFHHVSIAFLELRSSYREIIEDVTMRMGAGMAKYICKVVETIDDYDEYCHYVAGLVGLGLSRLFHASGVEILATDSLSESAGLFLQKTNIIRDYLQDINEIPKARMFWPHQIWSKYANKLEDFKYKENSMKAVQCLNEMVTNALLHVEDCLKLMSDLRDPAVLRSCAIPQIMAMGTLALCYNNIQVFRGAVKMRRGLGAKIFDQTRTMSDVYGAFCDFSSILMSKVNNSDPNATATLSTLEAIQKTCMESGNLTKRKSYIIEGKPSYNSALVSLYAAF
	Ese19G001283.t1	MLAGGTIPGLDELWAAFSYCIQQVRNYDYHHYLCLLELPPDMRRAAFALRSFNVETVRAMDVASVPKIGLMRLLWWQEATDKILANKLTENPTTQALASVISEHKVSVC
Panax ginseng	Pg_S5486.5	MSVALLGVVSHNYEISNVFGSLENAREGSRQLDSSRLISRNKNLLYTGRLKKGEIHKWSSCSLNAELRYSCLGCSGLENGSKYPIHSSMVVNPAGEMTVSSEQKVYDVVLKQAALVKKQLRSKEDLDVKPDIILPGTLSLLSEAYDRCGEVCAEYAKTFYLGTLLMTPERRRAIWAIYVWCRRTDELVDGPNASHITPSALDRWESRLDDLFKGRPFDMLDAALSDTVTKFPVDIQPFKDMIDGMRMDLKKSRYKNFDELYLYCYYVAGTVGLMSVPIMGIAPDSQATTESVYNAALALGLANQLTNILRDVGEDARRGRVYLPQDELAQAGLSNEDIFAGKVTDKWRNFMKKQIKRARMFFDEAEKGVTELSSASRWPVWASLLLYRQILDEIEANDYNNFTKRAYVSKPKKILALPVAYAKASRAGSPMVKAP
	Pg_S8673.1	MSVALLGVVSHNYEISNVFGSLENAREGSRLLDSSRLISRDKNLLCTGRLKKGEIHKWSSCSVNAELRYSCLGSSGLENGSKYPIHSSMVVNPAGEMTVSSEQKVYDVVLKQAALVKKQLRSKEDLDVKPDIILPGTLSLLSEAYDRCGEVCAEYTKTFYLGTLLMTPERRRAIWAIYVWCRRTDELVDGPNASHITPSALDRWESRLDDLFKGRPFDMLDAALSDTVTKFPVDIQPFKDMIDGMRMDLKKSRYKNFDELYLYCYYVAGTVGLMSVPIMGIAPDSQATTESVYNAALALGLANQLTNILRDVGEDARRGRVYLPQDELAQAGLSNEDIFAGKVTDKWRNFMKKQIKRARMFFDEAEKGVTELSSASRWPVWASLLLYRQILDEIEANDYNNFTKRAYVSKPKKILALPVAYAKASRATGKGTINIKF
	Pg_S3878.16	MSVALVWVVSPNSEISNCFGYLETGREGSRLLDSSRLISRDRNVNSGRLKKGKIQRWSSCALNAELRYSCLGSSRLQNESKFTVHSNMVANPTGEMTLSSEQKVYDVVLKQAALVKRQLRSSEDLEVKPDIILPGTLSLLSEAYDRCGEVCAEYAKTFYLGTLLMTPERRRAIWAIYVWCRRTDELVDGPNASHITPSALDRWELRLEDLFKGRPFDMLDAALSDTVTKFPVDIQPFKDMIDGMRMDLKKSRYKNFDELYLY

Species	Gene ID	Protein sequence
		CYYVAGTVGLMSVPIMGIALDSQATTESVYNAALALG<br>IANQLTNILRDVGEDARRGRVYLPQDELAQAGISNEDI<br>FVGKVTDKWRNFMRKQIKRARMFFDEAEKGVTELSP<br>ASRWPVWASLLLYRQILDEIEANDYNNFTKRAYVSKP<br>KKILALPAAYAKSLVAPSRTASPSLKA
	Pg_S2035.18	MSVALVWVVSPNSEISNCFGYLETGREGSRLLDSSRLM<br>SRDRNLNSGRLKKGKIQRWSSCALNAELRYSCLGSSR<br>LQYESKFTVHSSMVANPTGEMTLSSEQKVYDVVLKQ<br>AALVKRQLRSSEDLEVKPDIILPGTLSLLSEAYDRCGEV<br>CAEYAKTFYLGTLLMTPERRRAIWAIYVWCRRTDELV<br>DGPNASHITPSALDRWELRLEDLFKGRPFDMLDAALS<br>DTVTKFPVDIQPFKDMIDGMRMDLKKSRYKNFDELYL<br>YCYYVAGTVGLMSVPIMGIALDSQATTESVYNAALAL<br>GIANQLTNILRDVGEDARRGRVYLPQDELAQAGISNED<br>IFVGKVTDKWRNFMRKQIKRARMFFDEAEKGVTELSP<br>ASRWPVWASLLLYRQILDEIEANDYNNFTKRAYVSKP<br>KKILALPAAYAKSLVAPSRTASPSLKA
	Pg_S1881.17	MSSTLISVAAKPCIENQSFLFHKSTRVEATIAAPKRKSSS<br>TIFPELSVQSIPHTDLHVQEIVKRQTKRFVVDDDSCRKP<br>QFRPAFLEEAYEQCKNICSEYAKTFYLGTLLMTEERQK<br>AIWAIYVWCRRTDELVDGPNAAYMSSAVLDRWEERLY<br>DIFNGRPFDMLDAALAHTVHKFPLDIKPFADMIEGMR<br>MDTTKTRYKNFQELYLYCYYVAGTVGLMSVPVMGIPP<br>ESPVSAQSIYDAALYLGIGNQLTNILRDVGEDTLRGRIY<br>LPQDELTQYGLTDEDVFSRKVSNKWRQFMKEQITRAR<br>YYFNQAEEGASELDKDSRWPVWSSLMLYRNILDAIED<br>NEYDNLTKRAYVGRTKKLLMLPLAYSKSLSISSLMLQ<br>NQPIW
	Pg_S6893.7	MSSTLISVAAKPCIENQSFLFHKSTRVEATIAAPKRKSSS<br>TIFPELSVQSIPHTDLHVQEIVKRQTKRFVVDDDSCRKP<br>QFRPAFLEEAYEQCKNICSEYAKTFYLGTLLMTEERQK<br>AIWAIYVWCRRTDELVDGPNAAYMSSAVLDRWEERLY<br>DIFNGRPFDMLDAALAHTVHKFPLDIKPFADMIEGMR<br>MDTRKTRYKNFQELYLYCYYVAGTVGLMSVPVMGIPP<br>ESPVSAQSIYDAALYLGIGNQLTNILRDVGEDTLRGRIY<br>LPQDELTQYGLTDEDVFSRKVSNKWRKFMKEQITRAR<br>YYFNRAEEGASELDKDSRWPVIT
	Pg_S6162.3	MSGASSSGGLRSAFSYCVQQVRNYDYHHYLCLLELPP<br>DMRRAAFALRAFNVETARAMDVASDPKIGLMRLLWW<br>QDAIDKIFANKLIEHPTARALASVISEHKVSKSWLKRS<br>VEARINDAQREVSDIPQTVNDLEKYAEDTVSTILYTTL<br>QAGGIRSTTADHAASHIGKASGVLLLLKSLPYHVSRNR<br>HFSYIPAEVAGKHGLLVKQGGQSEIHMDSREGLCDAVF<br>EIATVANAHLQKARELAGTVPAEARPVLLPAVPAQVLL<br>DSLRRVHFDVFDPRLSHGILGSPPLLFQLKLKWHSWR<br>GKY
	Pg_S5036.7	MSGASSSGGLRSAFSYCVQQVRNYDYHHYLCLLELPP<br>DMRRAAFALRAFNVETARAMDVASDPKIGLMRLLWW<br>QDAIDKIFANKLIEHPTARALASVISEHKVSKSWLKRS<br>VEARINDAQREVSDIPQIVNDLEKYAEDTVSTILYTTLQ<br>AGGIRSTTADHAASHIGKASGVLLLLKSLPYHASRNRH<br>FSYIPAEVAGKHGLLVKQGGQSEIHMDSREGLCDAVFE<br>IATVANAHLQKARELAGTVPAEARPVLLPAVPAQVLLD<br>SLRRVHFDVFDPRLSHGILGSPPLLFQLKLKWHSWR<br>GKY
	Pg_S1678.33	MGSLGAILKHPDDFYPLLKLKIAARHAEKQIPSEPHWA

Species	Gene ID	Protein sequence
		FCYSMLHKVSRSFGLVIQQLGPQLRDAVCIFYLVLRAL<br>DTVEDDTSISTEVKVPILMAFHRHIYDNDWHFSCGTKE<br>YKVLMDEFHHVSNAFLDLGSGYKEAIEDITMRMGAG<br>MAKFICKEVETIDDYDEYCHYVAGLVGLGLSKLFHAS<br>GAEDLATDSLSNSMGLFLQKTNIIRDYLEDINEIPKSRM<br>FWPRQIWSKYVDKLEDLKYEENSGKAVQCLNDMVTN<br>ALLHVEDCLKYMSDLRDPAIFRFCAIPQIMSIGTLALC<br>YNNIQVFRGVVKMRRGLTAKVIDRTNTMSDVYGAFF<br>DFSCMLKSKVDNNDPNATKTLSRLEAIQKICKNSGALT<br>TKRKSYIIENESGYNSTLIVILFIILAILYAYLSSNLPNSL
	Pg_S2014.27	MGSLGAILKHPDDFYPLLKLKFAARHAEKQIPPEPHW<br>AFCYSMLHKVSRSFGLVIQQLGPQLRDAVCIFYLVLRA<br>LDTVEDDTSIPTEVKVPILMAFHRHIYDKDWHFSCGTK<br>EYKVLMDEFHHVSNAFLELGSGYQEAIEDITMRMGA<br>GMAKFICKEVETIDDYDEYCHYVAGLVGLGLSKLFHA<br>SGAEDLATDSLSNSMGLFLQKTNIIRDYLEDINEIPKSR<br>MFWPCQIWSKYVDKLEDLKYEENSAKAVQCLNDMV<br>TNALVHAEDCLKYMSDLRDPAIFRFCAIPQIMAIGTLA<br>LCFNNTQVFRGVVKMRRGLTAKVIDRTKTMSDVYGA<br>FFDFSCLLKSKARSNSENMQGVWNPVQKEIIHNRERV<br>RTQFSPDCYYLHYTSYPLCISIFKPTTEQTVRTTVHD
	Pg_S1637.7	MGSLGAILKHPDDFYPLLKLKIAARHAEKQIPSEPHWA<br>FCYSMLHKVSRSFGLVIQQLGPQLRDAVCIFYLVLRAL<br>DTVEDDTSISTEVKVPIVMAFHCHIYDNDWHFSCGTK<br>EYKVLMDEFHHVSNAFLDLGSSYKEAIEDITMRMGAG<br>MAKFICKEVETIDDYDEYCHYVAGLVGLGLSKLFHAS<br>GAEDLATDSLSNSMGLFLQKTNIIRDYLEDINEIPKSRM<br>FWPRQIWSKYVDKLEDLKYEENSGKAVQCLNDMVTN<br>ALLHVEDCLKYMSDLRDPAIFRFCAIPQIMAIGTLALC<br>YNNIQVFRGVVKMRRGLTAKVIDRTNTMSDVYGTFFD<br>FSCMLKSKVDNNDPNATKTLSRLEAIQKICKNSGALTT<br>KRKSYIIENESGYNSTLIIILFIILAILYAYLSSNLPNSL
	Pg_S0992.8	MGSLGAILKHPEDFYPLLKLKFAARHAEKQIPPEPHWA<br>FCYSMLHKVSRSFGLVIQQLGPQLRDAVCIFYLVLRAL<br>DTVEDDTSIPTEVKVPILMAFHRHIYDKDWHFSCGTKE<br>YKVLMDEFHHVSNAFLELGSGYQEAIEDITMRMGAG<br>MAKFICKEVETINDYDEYCHYVAGLVGLGLSKLFHAS<br>GAEDLATDSLSNSMGLFLQKTNIIRDYLEDINEIPKSRM<br>FWPRQIWSKYVDKLEDLKYEENSAKAVQCLNDMVTD<br>ALVHAEDCLKYMSDLRGPAIFRFCAIPQIMAIGTLALC<br>FNNTQVFRGVVKMRRGLTAKVIDQTKTMSDVYGAFF<br>DFSCLLKSKVDNNDPNATKTLSRLEAIQKTCKESGTLS<br>KRKSYIIESESGHNSALIAIIFIILAILYAYLSSNLLLNKQ
	Pg_S5881.8	MYDAALSDTVSTYPVDIQPFKDMIDGMRMDLKKSRY<br>QTFDELYLYCYYVAAGTVGLMSVPVMGIAPESKTSTE<br>NVYNAALALGIANQLTNIYIYIHFARRGRIYLPQDELAL<br>AGLTPDYIFRGKVTDKWRSFMRGQLKRARMFFDEAE<br>KGVAELSSVSRWPVWASLLLYWQILDAIEANDYDNFT<br>KRAYA
Polygala tenuifolia	Pt18G00871	MSLLLLKVCPNVEVFNTFGLFDSIRDVKLSDPSKVIPQ<br>DWGSIRPKRNVKERWSSFSCGANMYYSCIGEKRRQG<br>ASGFQVLPSMVANPAGEVAISSEQKVYDVVLKQASLV<br>NRQLRFPLDLDVKPDIVLPGSRDVLTEAYDRCGEICAE<br>YAKTFYLGTLLMTPERRRAIWAIYVWCRRTDELVDGP<br>NASHITPTALDRWESRLEDLFQGRPFDMLDAALSDTV<br>AKFPVDIQPFKDMIEGMRMDLRKSRYKNFDELYLYCY

Species	Gene ID	Protein sequence
		YVAGTVGLMSVPIMGIAPESQATTESVYNAALALGIANQLTNILRDVGEDARRGRIYLPQEELARAGLSEDDIFAGKVTDKWRIFMKNQIKRARKFFDEAENGITDLDAASRWPVWASLLLYRRILDEIEANDYNNFTKRAYVNKAKKLLFLPVSYGKSLITPSTKLSHSMKA
	Pt14G00460	MSLVLLWKVCPNAEAFNSFGLFDSSRDGKLLDSSKLIPQDWGSIKPKKNVKERWSSFSCGANTKYSCIGEKKRQGASRFQVLSSMVANPAGEIAVSSEQKVYDVVLKQASLVNRQLRSTVDLDIKPDIVLPGSRGVLSEAYDRCGEICAEYAKTFYLGTLLMTPERRRAIWAIYVWCRRTDELVDGPNASHITPTALDRWESRLEGLFEGRPFDMLDAALSDTVAKFPVDIQPFKDMIEGMRMDLKKSRYKNFDELYLYCYYVAGTVGLMSVPVMGIAPESQATTESVYNAALALGIANQLTNILRDVGEDARRGRIYLPQDELAQAGLSEDDIFAGKVTDKWRVFMKNQIKRARKFFDEAEKGVTELDAASRWPVWASLLLYRQILDEIEANDYNNFTKRAYVNKAKKLLFLPVSYGKSLITPSRKLSHSMRA
	Pt8G00157	MMSAVLHSVTCPSKEDANSSLSILDRSGTPNRKQKVLWSRMSMSSGISAFSSAVANPLRSSEERVYEVVLKQAALVKEQKRALPLNKPSEADHTAVKDLLNEAYERCGEVCAEYAKTFYLGTLLMTPESRRAVWAIYVWCRRTDELVDGPNASHITPKALDKWEQRLADLFEGRPYDMYDAALSDTVSKYPIDIQPLKDMIEGMRVDLRKSRYNSFDELYLYCYYVAGTVGLMTVPVMGIASESKASTQMVYNAALALGIANQLTNILRDVGEDARRGRIYLPQDELAQSGLSDEDVFRGKVTDKWRSFMKGQIKRARMFFDEAEEGVAELNGASRWPVWASLLLYKQILDAIEANDYDNFTKRAYVGKAKKLISLPVSYARSLVSWNR
	Pt10G01251	MSLPFSIAAKPSITGSMEFHCRKLTVRNSKVVINSLLRQRAARISPELSAQGVSLADLRIKEVVEKQSQKVKVTPSPQRKAPFQPNFLNEAYERCRKICAEYAKTFYLGTLLMTEERRKAIWAIYVWCRRTDELVDGPNADCITSAAALDRWEQRLEDIFYGHPYDMLDAAVTDTVAKFPLDIKPFRDMIEGMRMDTRKCRYENFQDLYLYCYYVAGTVGLMSVPIMGIAPESLVQAENIYSAALYLGIGNQLTNILRDVGEDASRGRVYLPQDELARFGLCDKDVLSSIITDKWREFMKQQITRARFYFNLAEEGASQLDKDSRWPVWSSLILYRKILDAIEENDYDNLTKRAYVGTAEKFLMLPLAYSKSISTHSSHFVRIPTK
	Pt8G00112	MTEERRKAIWAIYAWCRRTDELVDGPNADYMTAAVLERWEERLEDIFDGRPYDMLDAALTETVAKFPLDIKPFKDMIEGMRMDTRKCRYENFQELYLYCYHVAGTVGLMSVPIMGIAPESPIPAQDIYNAALYLGIGSQLTNILRDVGEDASRGRVYLPQDELAQFGLCDKDVFSRIVTDTWKEFMNEQIRRARFYFNLAEEGASQLDKDSRWPVWSSLIMYCKILDAIEDNDYDNLTKRAFVKWPKIFLALPLAYSRSI
	Pt9G01540	MGSLGAVLKHPDDFYPLLKLKLAVRHAEKQIPHEPHWGFSYSMLHKVSRSFALVIQQLDTDLRNAVCIFYLVLRALDTVEDDTSVPTDVKVPILKAFHRHIYDREWHFSCGTKEYKVLMDQFHHVSTAFLELDKGYQEAIEDITQRMGVGMAKFICKEVETVDDYDEYCHYVAGLVGLGLSKLFHASSKEDLASDNLSNSMGLFLQKTNIIRDYLEDINEIPKSRMFWPRQIWSKYVNKLEDLKHEENSDKAVQCLNDMVTNALMHAEDCLKYMSALKDLAIFRFCAIPQIMAIGTLALCYNNVQVFRGVVKMRRGLTAKVIHQTNTMADVYGAFYDFSCMLKSKVDRNDPNATKTLSRLESIQRTCLDS

Species	Gene ID	Protein sequence
		RLINKRKSFVNEGQPTYSSSMVIILFIVLAIILAYLTANPLNS
Arabidopsis thaliana	AT5G17230.3	MSSSVAVLWVATSSLNPDPMNNCGLVRVLESSRLFSPCQNQRLNKGKKKQIPTWSSSFVRNRSRRIGVVSSSLVASPSGEIALSSEEKVYNVVLKQAALVNKQLRSSSYDLDVKKPQDVVLPGSLSLLGEAYDRCGEVCAEYAKTFYLGTLLMTPERRKAIWAIYVMLKVDFYKQSIVALVWCRRTDELVDGPNASHITPMALDRWEARLEDLFRGRPFDMLDAALADTVARYPVDIQPFRDMIEGMRMDLKKSRYQNFDDLYLYCYYVAGTVGLMSVPVMGIDPKSKATTESVYNAALALGIANQLTNILRDVGEDARRGRVYLPQDELAQAGLSDEDIFAGKVTDKWRNFMKMQLKRARMFFDEAEKGVTELSAASRWPVWASLLLYRRILDEIEANDYNNFTKRAYVGKVKKIAALPLAYAKSVLKTSSSRLSI
	AT1G62730.1	MNGGGSSSLRSALSYCVQQVRNYDYHHYLCLLELPTEMRKAAFALRAFNVETARAMDVASDPKIGLMRLLWWQEAIDKLYTKKPINHPAAQALSWAISEHNISKPWLKRSVDARIRDAQREVDDIPESIAELEKYAEDTVSTLLYNTLQAGGISSTTADHAASHIGKASGLVLLLKSLPYHCTRNRHQSYIPADLAEKHGLLVKQGGRLEILLDNDSREGLSNVVFEIASVANAHLLKARELAGKVPAEAKPVLLHSVPVQVLLDSLNKVQFDVFDPRIQRGVLGVPPLLFQFKLKWYSWRAMF
	AT4G34640.1	MGSLGTMLRYPDDIYPLLKMKRAIEKAEKQIPPEPHWGFCYSMLHKVSRSFSLVIQQLNTELRNAVCVFYLVLRALDTVEDDTSIPTDEKVPILIAFHRHIYDTDWHYSCGTKEYKILMDQFHHVSAAFLELEKGYQEAIEEITRRMGAGMAKFICQEVETVDDYDEYCHYVAGLVGLGLSKLFLAAGSEVLTPDWEAISNSMGLFLQKTNIIRDYLEDINEIPKSRMFWPREIWGKYADKLEDLKYEENTNKSVQCLNEMVTNALMHIEDCLKYMVSLRDPSIFRFCAIPQIMAIGTLALCYNNEQVFRGVVKLRRGLTAKVIDRTKTMADVYGAFYDFSCMLKTKVDKNDPNASKTLNRLEAVQKLCRDAGVLQNRKSYVNDKGQPNSVFIIMVVILLAIVFAYLRAN
	AT4G34650.1	MGSLSTILRHPDELYPLLKLKLAITKAQKQIPLEPHLAFCYSILHKVSKSFSLVIQQLGTELRNAVCVFYLILRALDTVEDDTSVPVEIKVPILIAFHRHIYDGDWHFSCGTKEYKLLMDQFHHVSAAFLKLEKGYQEAIEDITKRMGAGMAKFICKEVETIDDYDEYCHYAAGLVGLGLSKIFIASELEILTPDWKQISNSTGLFLQKTNIIKDYLEDINERPKSRMFWPREIWGKYVDKLEDFKNEEKATKAVQCLNEMVTNALNHVEDCLKSLASLRDPAIFQSCAIPQIVAIGTLALCYNNVQVFRGVVRLRRGLIAKVIDRTKTMDDVYGAFYDFSCMLQTKVDNNDPNAMKTLNRLETIKKFCKENGGLHKRKSYVNDETQSKAIFVVMFVLLLAIVVVYLKANQCK
Oryza sativa	LOC_Os09g38320.1	MMSTTTTSSAAGSPVCARRRQRVFVDVPRRRATSLARVEYAKMAPPPPPPCSVRAAGSNPIGCLEVAEPWSGAAPPPLPPLPGHLHVAAPAAEDDDDALAAAAAAVPSEQRVHDVVLKQAALAAAAPEMRRPAQLAERERVAGGLNAAFDRCGEVCKEYAKTFYLATQLMTPERRRAIWAIYVWCRRTDELVDGPNASHMSALALDRWESRLDDIFAGRPYDMLDAALSHTVATFPVDIQPFRDMIEGMRLDLTKSRYRSFDELYLYCYYVAGTVGLMTVPVMGISPDSRANTETVYKGALALGLANQLTNILRDVGEDARRGRIYLPMDELEMAGLSEDDIFDGRVTDRWRCFMRDQITRARAFFRQAEEGASELNQESRWPVWASLLLYRQILDEIEANDYNNFTK

Species	Gene ID	Protein sequence
		RAYVPKAKKIVALPKAYYRSLMLPSSVRHCSSLTSS
	LOC_Os06g51290.2	MAAITLLRSASLPGLSDALARDAAAVQHVCSSYLPNNKEKKRRWILCSLKYACLGVDPAPGEIARTSPVYSSLTVTPAGEAVISSEQKVYDVVLKQAALLKRHLRPQPHTIPIVPKDLDLPRNGLKQAYHRCGEICEEYAKTFYLGTMLMTEDRRRAIWAIYVWCRRTDELVDGPNASHITPSALDRWEKRLDDLFTGRPYDMLDAALSDTISKFPIDIQPFRDMIEGMRSDLRKTRYKNFDELYMYCYYVAGTVGLMSVPVMGIAPESKATTESVYSAALALGIANQLTNILRDVGEDARRGRIYLPQDELAEAGLSDEDIFNGVVTNKWRSFMKRQIKRARMFFEEAERGVTELSQASRWPVWASLLLYRQILDEIEANDYNNFTKRAYVGKAKKLLALPVAYGRSLLMPYSLRNSQK
	LOC_Os12g43130.1	MASSSSAAALWTAAPHPHGSCIRIHAIFHQRHQRRGRRPVVVASSVRPLQAASLAVATAPVAVASRRTAAEEAVYEVVLRQAALVEEATHRRGAGAPRWAEEDAVDWGLLLGDAYHRCGEVCAEYAKTFYLGTQLMTPERRKAVWAIYVWCRRTDELVDGPNSSYITPKALDRWEKRLEDLFEGRPYDMYDAALSDTVSKFPVDIQPFKDMIEGMRLDLWKSRYRSFDELYLYCYYVAGTVGLMTVPVMGIAPDSKASTESVYNAALALGIANQLTNILRDVGEDSRRGRIYLPLDELAEAGLTEEDIFRGKVTDKWRKFMKGQILRARLFFDEAEKGVAHLDSASRWPVLASLWLYRQILDAIEANDYNNFTKRAYVNKAKKLLSLPVAYARAAVAS
	LOC_Os06g01470.1	MGSTAAAGGNLRTALSYCVQQVRNYDYHHYLCLLHLPPAMRKAAFAFRAFNIETAKAMDVVSDPKTGLMRLLWWKDVVDKVFANKLVEHPVAQVLSSVVSEHKISKHWLKRSVEARINDANRDDYAIPETISELERYAEDTQSTILYMTLQAGGIQSTIADHAASHIGKASGLLLLLKALPHHVSKQGRIPYIPASIAEECGLLTREGGRSEVRMGDELPDAVFKVASVADAHLQKARELASSVPAEAIPVLLPGVPAQVLLDSLRRREFNVFDSRLSRGVHGISPLWYQIKLIWHSVRKKY
	LOC_Os03g59040.1	MGVLSRPEEVLPLVKLRVAAGRIKRQIPPEEHWAFAYTMLQRVSRSFALVIQQLGPDLRNAVCIFYLVLRALDTVEDDTSIPAAVKVPILKEFHRHIYNRDWHYSCGTKDYKLLMDKFRLVSTAFLELGQGYQEAIEEITRLMGAGMAKFICKEVETVDDYNEYCHYVAGLVGYGLSRLFHAGGTEDLASDSLSNSMGLFLQKINIIRDYLEDINEIPKSRMFWPREIWSKYVNKLEDLKYEENSEKAVQCLNDMVTNALSHAEDCLQYMSALKDHAIFRFCAIPQIMAIGTCAICYNNVNVFRGVVKMRRGLTARVIDETNTMSDVYTAFYEFSSLIESKIDNNDPNASLTRKRVDAIKRTCKSSCSLKRRGYDLEKSKYNSMLIMVVLLLVAIVLGMIYAK
	LOC_Os07g10130.1	MGVVGALLGPEAEALVRLRAAAWRLRREVAAATDDDEHWAFAYSMLHRVSRSFAIVIQQLGPDLRNAVCVFYLVLRALDTVEDDTSIPTEIKVPILQEFHRHIYNRDWHFSCGTKDCKILMDKFHYVLTAFLELGSGYQEAIEEITRRMGAGMAKFICKEVETVDDYDEYCHYVAGLVGIGLSRLFHAAGLEDLAPESLSNSMGLFLQKVNITRDYLEDINEIPKSRMFWPREIWSKYVDKLEDLKYENNSVKAVKCLNEMVTNGLIHTEDCLRYMSALKDITILRFCAIPQVMALATYALCYNNVNVFRGVVKLRRGLTARIINETNSMADVYTAFYEFSSLLAEKIDDNDPNASLTRKRVNAIKETCKSSGLLKIRGYDLDRPKRNPAMIMMLLLLLVAIFLGALYRR
Platycodon	PgSS3	MSVALMWVVSPNSELSNGSLFMEMAREGNRLTDSSRF

Species	Gene ID	Protein sequence
grandifloru m		LARERNLFCHGRINNRKKKHKLNSCYLNADLKYSCLG DSGLESGKRFSVVSSMVANPAGEMTLSSEQLVYDVVL KQAALVKQLRSSRDTEVKPELILPGTLSVLSEAYDRCG EVCAEYAKTFYLGTLLMTPERRKAIWAIYVWCRRTDE LVDGPNASHITPKALDRWESRLEDLFNGRPFDMLDAA LSDTVAKFPVDIQPFKDMIEGMRMDLRKSRYKNFDEL YLYCYYVAGTVGLMSVPIMGIAPDSQATTESVYNAAL ALGIANQLTNILRDVGEDARRGRVYLPQDELAQSGLS DADVFAGKVTDKWRHFMKKQIKRARMFFDEAEKGV TQLSSASRWPVWASLLLYRQILDEIEANDYNNFTRRAF VSKQKKIVALPIAYAKALVSPTSRKSSPLAKA
	PgSS4	MSSAFSMAAQPCYIGVRVSNGKLVSHNKVTTTAAIAA PKRSNGAIVAPELSIKGIPHTDLKVREIVQRQSRTQSFFT DDHACGKPRFHPEFLDEAYERCRNICAEYAKTFYLGT RLMTEERQKAIWAIYVWCRRTDELVDGPNAAHMSSA VLDRWEERLDDIFNGRPFDMLDAALTDTVHNFPLDIK PFRDMIEGMRMDTRKSRYENFQELYLYCYYVAGTVGL MSVPVMGISPESPVSAQSIYNSALYLGIGNQLTNILRDV GEDAQRGRIYLPQDELAQFGLCDEDVFSRTVSGRWRE FMKEQINRARFYFNQAEEGASQLDKDSRWPVWSSLLL YREILDAIEENDYDNLTKRAYVGRTKKLLMLPLSYTRA LATPSLAFP
	PgSS1	MNGASTSSGLRSAFSYCVQQVRNYDYHHYLCLLELPT NMRRAGFALRAFNIETARAMDVASDPKIGLMRLLWW KEAVDKIFMNNLIEHPTAQALASVVSEQKISKSWLKRS VEARINDAQREVTEIPETIAELEKYAEDTVSTILYTTLQ AGGIRSTAADHAASHIGKASGLLLLLKSLPYHANRQR QFSYIPAEVAEKHGLLVKQGGRSEIHTDSREGLSNAVFE IASVAFVHLQKARELAGTVPAEARAVLLPAVPAQVLLD SLSDAQFDVFDSRLNRGILGVPPLWFQLKLKWHSWR GKY
	PgSS2	MGSLGAILKHPDDFYPLLKLKMAAKKAEKQIPPEPHW GFCYSMLHKVSRSFALVIQQLGTELRDAVCIFYLVLRA LDTVEDDTSIETEVKVPILIAFHRHIYDRDWHFACGTK EYKVLMDQFHHVSAAFLELGSSYQEAIEDITMRMGAG MAKFICKEVETVDDYDEYCHYVAGLVGLGLSKLFHAS GKEILFPDSLSNSMGLFLQKTNIIRDYLEDINEIPKSRMF WPRQIWSKYVNKLEDLKYEENSTKAVQCLNDMVTNA LIHVEDCLKYMSDLRDPAIFKFCAIPQIMSIGTLALCYN NIEVFRGVVKMRRGLTAKVIDRTKTMADVYGAFYDFA SMLKSKVDMNDPNATKTLSRLDAIQKTCRDSGTLAKR KSYIIESDAKYNSTLIIVLFIILAILYAYLSTNRPNSTRKSF QCDFVSTL

Table S3 Protein sequences of *SE* gene families in six different plant species

Species	Gene ID	Protein sequence
Eleutheroco ccus	Ese07G002519.t1	MLMGIRTPTYPFSFSLQQHTKSRPPRARIRAGHTKLNN PSNPITINNDNTEVERKRQQQQDLTVLSSSSSGPQLYFY

Species	Gene ID	Protein sequence
senticosus		TKKMGIMMDTSYCIAGTLFASLLGFSFLYILGHHNSTI<br>ANSKKKNQQQKMTAARSVVTRNDPNNPWGNGSDAD<br>IIIVGAGVAGAALAYTLGKDGRRVHVIERDLTEPDRIVG<br>ELLQPGGYLKLIELGLEDCVEEIDAQRVIGYALFKDGR<br>NVKLSYPLEKFHSDVSGRSFHNGRFIQRMREKAARLP<br>NVRLEQGTVSSLLEENGTIKGVQYKIKTGQKVKAYAP<br>LTIVCDGCFSNLRRSLCKPNVDVPSCFVGLILENCKLPH<br>PNHGHVILADPSPILFYPISSTEVRCLVDIPGQKLPSLAN<br>GEMATYLKTMVAPQIPPELHDAFMAAINKGNIRTMPN<br>RSMPAAPYPTPGALLMGDAFNMRHPLTGGGMTVALS<br>DIVVLRNLLKPLRDMNDADSLCKYLESFYTLRKPVAS<br>TINTLAGALYKVFCASPDQARKEMREACFDYLSLGGV<br>CSTGPMALLSGLNPQPLSLVLHFFAVAIFGVGRLLLPFP<br>SPKRLWNGVRLISVASGIIFPIIQAEGVRKMFFPATLPAY<br>YRAPPADDIKF
	Ese19G001396.t1	MELGRSYVVIIEPYLIFTATFLFGFVLLFTLRWKREKKG<br>GAVSMGVNGAYKMTSSSELNGHCSPDQDIDGCSADVI<br>IVGAGVAGSALAYTLAKDGRRVHVIERDLTEQDRIVGE<br>LLQPGGYLKLVELGLEDCVNEIDAQRVFGYALYMDGK<br>NTRLSYPLEKFHADVAGRSFHNGRFIQRMREKAASLP<br>NVRMEQGTVTSLVEKEGIVKGVQYKAKNGQEMCAYA<br>PLTIVCDGCFSNLRRSLCNPKVDVPSCFVGLILENIDLP<br>HINHGHVILADPSPILFYKISSTEIRCLVDVPGQKVPSIA<br>NGELAHYLKTSVAPQIPPELYKSFIAAIDKGQIKTMPNR<br>SMPADPHPTPGALLLGDAFNMRHPLTGGGMTVALSDI<br>VLIRDLLRPLRNLHDSSTLCSYLESFYTLRKPVASTINT<br>LAGALYKVFCASPDQARQEMRDACFDYLSLGGICSEG<br>PIALLSGLNPRPMSLFFHFFAVAIYGVGRLLIPFPSPKKI<br>WLGARLISGASGIIFPIIKSEGVRQMFFPATVPAYYRAPP<br>FTKEM
	Ese22G002027.t1	MELGRSYMVNDQYFLGGISVATFLFGFVVLFALRRKR<br>EKKGGATSMAIDGTYKNNIKMTAGSEVNGHCSLEDVA<br>GSSDDVIIVGAGVAGSALAYTLAKDGRRVHVIERDLTE<br>QDRIVGELLQPGGYLKLVELGLEDCVNEIDAQRVFGY<br>ALYMYGKNTRLSYPLEKFHADVAGRSFHNGRFIQRMR<br>EKAASLPNVRMEQGTVTSLVEKKGTVKGVQYKTKNG<br>QVMSAYAPLTIVCDGCFSNLRRSLCNPKVDVPSCFVGL<br>ILENVDLPHINHGHVILADPSPILFYKISSTEIRCLVDVP<br>GQHVPSIANGELAHYLKTSVAPQIPPELYKSFIAAIDKG<br>QIKTMPNRSMPADPHPTPGALLLGDAFNMRHPLTGGG<br>MTVALSDIVLIRDLLRPLRDLHDSSTLCKYLESFYTLR<br>KPVASTINTLAGALYKVFCASPDQARQEMRDACFDYL<br>SLGGICSEGPIALLSGLNPRPMSLFFHFFAVAIYGVGRLL

Species	Gene ID	Protein sequence
		IPFPSPKKMWLGARLISIWILVAESYGEDCTSVCMGRR<br>NKRRKNKMEPKNHSVLSQCFLDTLSPHPEAVQNPASL<br>PPPIP
	Ese24G001061.t1	MNSSSSTLSSSTETLHSFMEAMLIDLYILGWIFAFLFGF<br>LLLFNFNRKREKINGANSTEFGTDSINNTSSGNGYYRP<br>ENIAGSTDVIIVGAGVAGSALACTLAKDGRRVHVIERD<br>LSEQDRIVGELLQPGGYLKLIELGLEDCVNEIDAQRVF<br>GYALYMDGKNTRLSYPLEKFHADVAGRSFHNGRFVQ<br>RMREKASSLPNVRMEQGTVTSLVEKKGTVKGVQYKT<br>KDGQELSAFAPLTIVCDGCFSNLRRSLCNPKVEVPSCF<br>VGLILENIDLPHINHGHVILADPSPILFYKISSTEIRCLVD<br>VPGQKVPSISNGELAHYLKTVVAPQVPRELYNSFIAAV<br>DTGNIRTMPNRSMPADPHPTPGALLLGDAFNMRHPLT<br>GGGMTVALSDIVLIRDLLRPLRDLHDSSTLSKYLESFY<br>TRRKPVASTINTLAGALYNVFCASPDKARQEMRNACF<br>DYLSLGGICSQGPIALLSGLNPRPLSLFLHFFAVAIYGV<br>GRLLIPFPSPKRMWLGARLISGASGIIFPIIKSEGVRQMF<br>FPATVPAYYRAPPIH
	Ese16G002000.t1	MNSSSSTLSSSSTTDKLHSFMEAAILVDQYYLGWIFAF<br>LFGFLMLFNFKRKREKSGANSTDFGTDSIINTSSENGY<br>NSPEHIAGSTDVIVVGAGVAGSALAYTLAKDGRRVHVI<br>ERDLTEQDRIVGELLQPGGYLKLIELGLEDCINEIDSQR<br>VFGYALYMDGKNTRLPYPLEKFHADVAGRSFHNGRFV<br>QRMREKAASIPNVRMEQGTVTSLVEKKGTIKGVQYKT<br>KDGQELSAFAPLTIVCDGCFSNLRRSLCNPKVEVPSCF<br>VGLILEDIDLPYINHGHVILADPSPILFYKISSTEIRCLVD<br>VPGQKVPSISNGELANYLKTVVAPQVPKELYNSFIAAV<br>DKGNIKTMPNRSMPADPHPTPGALLLGDAFNMRHPLT<br>GGGMTVALSDIVLIRDLLRPLRDLRDSSTLCKYLESFY<br>TLRKPVASTINTLAGALYKVFCASPDKARQEMRNACF<br>DYLSLGGICSQGPIALLSGLNPRPVSLFLHFFAVAIYGV<br>GRLLIPFPSPTRMWLGARLISGASGIIFPIIKSEGVRQMF<br>FPATVPAYYRAPPMHSLRK
	Ese01G001295.t1	MEEHYALGSILASLLGFILVYALFFKKNDRRDSVEAAE<br>STTATTTAIKGECRSCNGAGDDADVIIVGAGVAGAAL<br>AHTLGKDGRRVHVIERDLTEPDRIVGELLQPGGYLKLI<br>ELGLEDCVEEIDAQKVFGYALFKDGKNTRLSYPLEKF<br>HSDVSGRSFHNGRFIKRMRDKAATLPNVQMEQGTVTS<br>LLEENGTIKGVQYKTKNGEEMSAYAPLTIVCDGCFSNL<br>RRNLCSPKVDVPSCFVGLVLENCELPHANHGHVILAD<br>PSPILFYPISSTEIRCLVDVPGQKVPSISNGEMATYLKTV<br>VAPQIPPELHDAFIATVEKGSIRTMPNRSMPAAPHPTPG<br>ALLMGDAFNMRHPLTGGGMTVALSDIVVLRNLLRPLR

Species	Gene ID	Protein sequence
		DMNDASTLCKYLESFYTLRKPVASTINTLAGALYKVF<br>CASPDQARKEMREACFDYLSLGGVCSEGPVSLLSGLN<br>PRPLSLVVHFFAVAIFGVGRLLLPFPSPKRMWIGARLIST<br>ASRIIVPIIKAEGFRQMFFPATVPAYYRAPPVW
	Ese03G001204.t1	MEEHYVLGSILASLLGFILVYALFAKKNARRDSVEAAK<br>STTATTTTAIKGECRSRNGAGDDADVIIVGAGVAGAAL<br>AHTLGKDGRRVHVIERDLTEPDRIVGELLQPGGYLKLI<br>ELELEDCVEEIDAQRVFGYALFKDGKNTRLSYPLEKFH<br>SDVSGRSFHNGRFIQRMRKKAATLPNVQMEQGTVTSL<br>LEENGTIKGVQYKTKTGEEMSAYAPLTIVCDGCFSNLR<br>RNLCCPKVDVPSCFVGLVLENCELPHANHGHVILADP<br>SPILFYPISSTEIRCLVDVPGQKVPSISNGEMATYLKTVV<br>APQIPPELHDAFIATVEKGNIRTMPNRSMPAAPHPTPGA<br>LLMGDAFNMRHPLTGGGMTVALSDIVVLRNLLRPLRD<br>MNDASALCKYLESFYTLRKPVASTINTLAGALYKVFC<br>ASPDEARKEMREACFDYLSLGGVYSEGPVSLLSGLNP<br>RPLSLVVHFFAVAIFGVGRLLLPFPSPKRMWIGARLISS<br>ASGIIFPIIKAEGLRQMFFPATVPAYYRAPPVWVVFVHS<br>GVEDREGWAGIRGTSDKSIDDLKLLLNYQKIKMASGN<br>QELNYIMHEENVAENQEATNGPAANSGNNGANNMMH<br>NDFFKNFMQFMQNQARRIPVADNDGNISNARLVTVKK<br>FKELGPLEFHGKPDPLKAEAWIKQITKIFDVLRFTDDQ<br>KVPFATFMLRGEADHWWESVKRTHRLALEMSWAEFQ<br>ELFNDKYFSESIRHMKEVEFIKLERGESGENNLKRKRF<br>HNDRGMAPTSAPTQASVPRGGDRPPVRCYKCGEEGHI<br>SPNCTQQSKVCYNCGKEGHMARFCPVTKSSPAAIMAP<br>KANTDKGKSVVRGSAFVVTSHGERYPNEVITGIDWLT<br>THHAKIDCFQKIISFHIPNQPVMQIRAAKPLKSVVVISS<br>HKAIRLLKNGCQVFLAHVTDLNKNTSDLNNIPIVNEFP<br>EELSGLPPNREIDFCIDLEPGTKPIFKAPYRMAPIELQEL<br>KVQLQELLDAGFIRPSTSPWGAPILFVKKKDGSMRLCI<br>DYRELNHVTIKNRYPLPRINDMFNQLTRYGHYEFLVM<br>PFGLTNAPTAFMDMMNRIFKEYLDHFVVVFIDDILIYS<br>KSREDHEQHLRLILQKLRENKLYAKLKKCEFWLEEVS<br>FLGHVISKQGISVDPAKIESILNWQQPKSVTEVRSFLGL<br>AGYYRRFVEGFSRIATPLTKLTWKSTKFEWSEECEKQE<br>LKRRLVSAPVLTIPSGSGGFVIYSVASGRGLGCVLMQH<br>RKVVAYASRQLKDYEKNYPTHDLELAAVVFALKIWRH<br>YLYGEKVEIFTDHKSLKYFFTQKELNMRQRRWLELL<br>KDYDHTIQYHPEKANVVADALSRRSYTSTNAMLTTQG<br>HILNDLQRMEIEMKTPGSHMRIAHVRVKPTLIDRIKAA<br>QATDPELMKMVKKVQEGAIPEARIDEKGILWVNSRLC<br>VPNNPELKHKAEHQVPKGPLQPLEVPEWKWEHINKD

Species	Gene ID	Protein sequence
		FVTGFPKVSQGLDAIWVIVDRLTKSAHFLPIREDYPLE<br>RLAREAKLLGPELVRLTTEKIQIIRQRIQSAQDRQKNYA<br>DLYRKDEIFNVGDNVFLRVSPWKGVTRFGRRGKLNPK<br>FVGPFEILDRVGTVAYRLALPPIMSRIHNVFHISMLRKY<br>VKDPNHVIELEPDVLAKDLTYEEVPVKILDRKEQILRS<br>KVIPQVKVLWRNQEVEEMTWELESEMKSKYPHLFETP<br>DDIQLVHLLVLSSFFNPLFNATELVIDAQTLLLLGVDDE<br>S
	Ese19G001421.t1	MTSYEFILGGAIVSFLGFAVFYLLAENRKSNNSSNLVQI<br>KRNECAKTAQDGECQQEGIGNPDIIIVGAGVAGSALAY<br>TLGKDGRRVLVVERDLSEPDRIVGELLQPGGYLKLIEL<br>GLEDCVNEIDSQQVFGYALFKDGKSTRLSYPLKEFHTD<br>VTGKSFHNGRFIQQMREKAATLSNVKLEQGTVTSLVE<br>RKGTVKGVHYKTKSGQDMTAYAPLTIVCDGCFSNLRR<br>SLCNPKVEIPSVFVALILKNCQLPYPNHGHVILANPSPIL<br>LYRISSTEIRCLVDVPGKKVPSIANGDMAHYLKTLVAP<br>QIPFELYDAFITAIDEGNMKTMANRSMPADPYPTPGAL<br>LLGDAFNMRHPLTGGGMTVALSDIVVLRDLLRPLGDL<br>NDAPALCRYLESFYTLRKPVASTINTLAGALYKVFCAS<br>PDQARNEMRQACFDYLSLGGIFSNGPVALLSGLNPRPL<br>SLVLHFFSVAIYGVGRLLLPIPSLKRAWLGARLISDASSI<br>IFPIIKAEGIRQMFFPRVMPAYYRCPPTN
	Ese14G000263.t1	MGKIESLESCPVVFGWLCYTRDGRRVHVIERDLTEPDR<br>IVGELLQPGGYLKLMELGVEDCVEEIDAQRVFGYALF<br>KDGKNTRLCYPTEKFHSDVSGRSFHNGRFIQRMREKA<br>ATLPNIKLEQGTVTSLLEENGTIKGVQYKTKTAEKMSV<br>YAPLTIVCDGCFSNLRNNLCSPKVDVPSCFVGLVLEKC<br>QLPYANYGHVILADPSPILFYPISSTEIRCLVDVPGQKVP<br>SISNGEMATYLKTVVAPQIPPELHDAFITTIDKGNIRTM<br>PNRSMPAFPHPTPGALLMGDAFNMRHPLTGGGMTVAF<br>SDIVVLCNLVRPLRDMNDASTLCKYLESFYTLRKPVAS<br>TINTLADALYKVFCASPDQARKEMREACFDYLSLGGV<br>CSEGPVSLLSGLKPRPLSLVVHFFAVAIFGVGRLLLPFPS<br>PKRMWNGARLISSALGIIFPIIKAEGVRQMFCPAMVPA<br>YYRAPPVM
	Ese24G001063.t1	MREKAATLSNVKLEQGIVTSLVERKGTVKGVHYKTKT<br>GQDMIAYGPLTFVCDGCFSNQRRSLCNPKVDIPSSFVS<br>LILKNCQLPYPNHGHVILANPSHILLYRISSTEIRCLVDV<br>LGKKVPSIANGDMIPFELYDAFIRAIDEGNMRTMANRS<br>MPATPYPTLGALLLGDAFNMRHPLTGGGMTVALSDIV<br>VFRDLLRLLTKQSRVLTKVGDFLITLASYCLQPVASTIK<br>TLTGALYKVFCASPDEARNEMRQAYFDYLSLGGIFSN<br>GPVALLSGLNPRPLSLVLHFFSVAMYGVGCWLLPFPSP

Species	Gene ID	Protein sequence
		KRAWPGARLIWDASSIIFPIIKAEGVKQMFFPAVIPAYY<br>RSPPTD
	Ese06G002592.t1	MPFDDSNEGGIDPILGASQLTWSIICHYLSNTEVRCLVD<br>VPGQKLPSLASSEMANYLKTMVAPQLPPELHDAFIAAT<br>DKGNTRTLPNRSMPAAAYSTPLALLMGCAFNICVILQL<br>VDWWTGGGMTLALSDIVVLRNLLKPLHDMTDADSLC<br>KYLESFYTLRKPVASTINTLAGALYKVFCASPDQARKE<br>MREACFDYVSLGGVFSTGPVALLSGLNSRPLSLVLHFF<br>AVAIYAVGCLLLPFPSPKRLWTGVRLISVSSCNLFKTGY<br>LVFDIMLHLLSCQALLPYS
	Ese05G001251.t1	MVNAGEVNAKLDALMAQQDERFQRLEDSMAALTGH<br>VQTSVTAIQASITALTARQGPNNFHAPPDPRLNAVDRG<br>AKLEVGDFNGDQGPEAFFDWVHSLESFFRWYNLTDET<br>KLFFADAKLKGTTCIWWDHHQRNLYTAARRWEDMK<br>AAMTRRFVPPDYKQKACLQLTELVQGSKSVAEYTSTF<br>YSLVTRSEFPWIEDVLISNRRGLNHEIASKVPVQTRSER<br>TFVEPLRSRSVERSSINSSATSRPNRTTGSSSASNNPSAR<br>CFNCRGFGHMSFECPSSKMNVATTPSRVAVVNKDSEK<br>TDVHEEVCPPSDEENEYNEQDLEADAHLEGCLGVLFD<br>LYWLLRSKRMFLNSIVGGYFRRELDVQVKYSSLVIDT<br>GSCTDVISEEAVKKLGLKVEPHPDPYDVAWITNTKLRV<br>SQRCLVTFSVGKLKDTVMCDILPLKVCHIILGRTWLW<br>DRHAHHDARANTYSIMKGSTKFILTCALDMLKLKLAK<br>NSLVDVPSCFVGLILENCQLPHANYGHVILADPSPILFY<br>PISSTEIRCLVDVPGQKVPSISNGEMATYLKTVVAPQLH<br>IDLCCSIFPDSPHVLLDAFIATIDRGNIRTVPNRSMPAAP<br>HPTPGALLMGNAFNMQHPLTGGGTTVALSDIVVLCNL<br>LRPLHDMNGACTLLSSNI
Panax ginseng	Pg_S1693.31	MGMMMDYIAGTLFAFLLGFSFLYILGTIANSKKKNQK<br>QKATACTSFETRNDLNDGDNPRANQSDADVIIVGAGV<br>AGAALAHTLGKDGRRVHVIERDLTEPDRIVGELLQPG<br>GYLKLIELGLEDCVEEIDAQRVIGYALFKDGRNTKLSY<br>PLEKFHSDVSGRSFHNGRFIQRMREKAATLSNVHLEQ<br>GTVSSLLEENGTIKGVQYKTKTVQDVKAYAPLTIVCD<br>GCFSNLRRSLCKPKVDVPSCFVGLILENCKLPHPNHGH<br>VILADPSPILFYPISSTEVRCLVDVPGQKLPSLANGDLA<br>NYLKTMVAPQIPPELHDAFIAAIDKGNIRTMPNRSMPA<br>APYPTPGALLMGDAFNMRHPLTGGGMTVALSDIVVLR<br>NLLKPLGDMNDADSLCKYLESFYTLRKPVASTINTLA<br>GALYKVFCASPDQARKEMREACFDYLSLGGVCSTGPV<br>ALLSGLNPRPLSLVLHFFAVAIYGVGRMLLPFPSPKRLW<br>AGVRLISAASGIIFPIIQAEGVRQMFFPATVPAYYYRAPP<br>ADDIKF

Species	Gene ID	Protein sequence
	Pg_S3064.5	MEQGTVTSLVEKKASVKGVQYKTKDGQELSAFAPLTI VCDGCFSNLRRSLCNPKVEVPSCFVGLILENIDLPHVN HGHVILADPSPILFYKISSTEIRCLVDVPGQKVPCISNGE LANYLKTVVAPQVPKQLYNSFIAAVDKGNIRTMPNRS MPADPHPTPGALLLGDAFNMRHPLTGGGMTVALSDIV LIRDLLRPLRDLHDSSTLCKYLESFYTLRKPVASTINTL AGALYKVFCASPDKARQEMRNACFDYLSLGGICSQGP IALLSGLNPRPISLFLHFFAVAIYGVGRLLIPFPSPKRMW LGARLILGASGIIFPIIKSEGLRQMFFPATVPAYYRAPPIH
	Pg_S2606.7	MELERSYRENDEYFLMFAATLLFGFVLYLFTLRRRRRR REKKGGAGSMEIINGAYKMTSSSEVNGHCTPEDIAGSS DDVIIVGAGVAGSALAYTLAKDGRRVHVIERDLTEQD RIVGELLQPGGYLKLVELGLEDCVNEIDAQRVFGYALY MDGKNTRLSYPLEKFHADVAGRSFHNGRFIQRMREK AASLPNVRMEQGTVTSLVEQKGTVKGVRYKTKNGQE MSAAYAPLTIVCDGCFSNLRHSLCNPKVDVPSCFVGLI LENIDLPHINHGHVILADPSPILFYKISSTEIRCLVDVP GQKVPSIANGELAHYLKTSVAPQIPPELYKSFIAAIDKG KIKTMPNRSMPADPHSTPGALLLGDAFNMRHPLTGGG MTVALSDIVLIRDLLRPLRDLHDSSTLCKYLESFYTLR KPVASTINTLAGALYKVFCASPDKARQEMRDACFDYL SLGGICSEGPIALLSGLNPRPMSLFFHFFAVAIYGVGRLL IPFPSPRKMWLGARLISGASGIIFPIIKSEGVRQMFFPAT VPAYYRAPPITKKM
	Pg_S6308.10	MNSSSSTTTTDTLHSFMEASALLIDQYFLGWIFAFLFGF LLLLNFKRKREKNNSTEFGTDDSNGYYTPENIAGSTD VIIVGAGVAGSALAYTLANDGRRVHVIERDLTEQDRIV GELLQPGGYLKLIELGLEDCVNEIDAQRVFGYALYMD GKNTRLSYPLEKFHSDVAGRSFHNGRFVQRMREKAAS LPNVRMEQGTVTSLVEKKGSVKGVQYKTKDGQELSA FAPLTIVCDGCFSNLRRSLCNPKVEVPSCFVGLILENID LPHINHGHVILADPSPILFYKISSTEIRCLVDVPGQKVPC ISNGELANYLKTVVAPQVPKQLYNSFIAAVDKGNIRTM PNRSMPADPHPTPGALLLGDAFNMRHPLTGGGMTVAL SDIVLIRDLLRPLRDLHDSSTLCKYLESFYTLRKPVASTI NTLAGALYKVFCASPDKARQEMRNACFDYLSLGGICS QGPIALLSGLNPRPISLFLHFFAVAIYGVGRLLIPFPSPKR MWLGARLILGASGIIFPIIKSEGLRQMFFPAIVPAYYRAP PIH
	Pg_S0129.28	MEEHYVLGLILASLLGFLLAYALFFKKNDRRYSSEAVK SNTATTTTAINGECRSRNGAGDDVDVIIVGAGVAGAAL AHTLGKDGRRVHVIERDLTEPDRIVGELLQPGGYLKLI ELGLEDCVEEIDAQRVFGYALFKDGKNTRLSYPLEKFH

Species	Gene ID	Protein sequence
		SDVSGRSFHNGRFIQRMREKAATLPNVQMEQGTVTSL LEENGTIKGVQYKTKTGEETSAYAPLTIVCDGCFSNLR RNLCSPKVDVPSCFVGLVLENCKLPHANHGHVILADP SPILFYPISSTEIRCLVDVPGQKVPSISNGEMATYLKTVV APQIPPELHDAFIATVEKGNIRTMSNRSMPAAPHPTPGA LLMGDAFNMRHPLTGGGMTVALSDIVVLRNLLRPLRD MNDASTLCKYLESFYTLRKPVASTINTLAGALYKVFC ASPDQARKEMREACFDYLSLGGFCSEGPVSLLSGLNP RPLSLVVHFFAVAIFGVGRLLLPFPSPKRMWIGARLISS ASGIIFPIIKAEGFRQMFFPATVPAYYRAPPVC
	Pg_S2840.6	MEEHYVLGLILASLLGFLLVYALFFKKNDRRYSVEAVK SNTATTTTAINGECRSRNGAGDDVDVIIVGAGVAGAAL AHTLGKDGRRVHVIERDLTEPDRIVGELLQPGGYLKLI ELGLEDCVEEIDAQRVFGYALFKDGKNTRLSYPLEKFH SDVSGRSFHNGRFIQRMREKAATLPNVQMEQGTVTSL LEENGTIKGVQYKTKTGEEMSAYAPLTIVCDGCFSNLR RNLCSPKVDVPSCFVGLVLENCKLPHANHGHVILADP SPILFYPISSTEIRCLVDVPGQKVPSISNGEMATYLNTVV APQIPPELHDAFIATVEKGNIRTMSNRSMPAAPHPTPGA LLMGDAFNMRHPLTGGGMTVALSDIVVLRNLLRPLRD MNDASTLCKYLESFYTLRKPVASTINTLAGALYKVFC ASPDQARKEMREACFDYLSLGGFCSEGPVSLLSGLNP RPLSLVVHFFAVAIFGVGRLLLPFPSPKRMWIGARLISS ASGIIFPIIKAEGFRQMFFPATVPAYYRAPPVW
	Pg_S3767.14	MDGKNTRLSYPLEKFHADVAGRSFHNGRFIQRMREK AASLPNVRMEQGTVTSLVEQKGTVKGVRYKTKNGQE MSAAYAPLTIVCDGCFSNLRHSLCNPKVDVPSCFVGLI LENIDLPHINHGHVILADPSPILFYKISSTEIRCLVDVPG QRVPSIANGELAHYLKTSVAPQIPPELYKSFIETIDKGQI KTMPNRSMPADPHPTPGALLLGDAFNMRHPLTGGGM TVALSDIVLIRDLLRPLRDLHDSSTLCKYLESFYTLRKP VASTINTLAGALYKVFCASPDKARQEMRDACFDYLSL GGIFSEGPIALLSGLNPRPMSLFFHFFAVAIYGVGRLLIP FPSPRKMWLGARLISGASGIIFPIIKSEGVRQMFFPATVP AYYRAPPITKKM
	Pg_S6152.1	MAISMDINMINESIFVGVLLVLVVLLLLGKRRNIHNNL VPIKIKRNECAKPPQDGESRPEIAENPDVIIVGAGVAGS ALACTLGKDGRRVLVIERDLTEQDRIVGELLQPGGYLK LMELGLEDCVDKIDAQEVFGYALFKDGKSTRLSYTLK DFHSDVAGRSFHNGRFIQRMREKAATLSNVRLEQGTV TSLIEKKGTVKGVHYRSKTGQELTACAPLTIVCDGCFS NLRRSLCNPKVEIPSHFVAMILKNCQLPYPNHGHVILA NPSPILLYRISSTEIRCLVDIPGQKVPSIGNGDMSHYLKT

Species	Gene ID	Protein sequence
		VVAPQIPLDLNKAFISAIEEGNIRTMANRSMPADPYPTP<br>GAILLGDAFNMRHPLTGGGMTVALSDIVVLRDLLRPL<br>RDFNDAPSLSKYIEAFYTLRKPVASTINILAGALYKVFC<br>ASPNQARNEMRQACFDYLSLGGIFSNGPIALLSGLNPQ<br>PFILVLHFFSVAIYGVGRLLLPLPSPKRAWHGARLILDA<br>SAIIFPIIKAEGVRQMFFPSTISAYHRSPPIY
	Pg_S4651.3	MELGLEDCVDKIDAQEVFGYALFKDGKSMSYPLKDF<br>HLDVAGRSFHNGRFIQRMREKAATLSNVRLEQGIVTSL<br>IEKKGTVKGVHYRTKTGQELTACAPLTIVCDGCFSNLR<br>RSLCNPKVEIPSHFVAMILKNCQLPYPNHGHVILANPSP<br>ILLYRVSSTEIRCLVDIPGQKVPSIGNGDMSHYLKTVVA<br>PQIPLDLNKAFISAIEEGNIRIMANRSMPADPYPTPGAL<br>LLGDAFNMRHPLTGGGMTVALSDIVVLRDLLRPLLDF<br>NDAPSLSKYLEAFYTLRKPVASTINTLAGALYKVFCAS<br>PNQARNEMRQACFDYLSLGGIFSNGPIALLSGLNPQPL<br>SLVLHFFSVAIFGVGRLLLPLPSPKRAWLGARLISDTSAI<br>IFPIIKAEGIRQMFFPSTISAYHRSPPTY
	Pg_S2606.8	MIVQHMLAGAIVSLLGFAVLYLLGKKNKSNNSALVQT<br>QGNECAEWQPEIIENPDIIIVGAGVAGSALACTLGKDG<br>RRVLVIERDLTEPDRIVGELLQPGGYLKLVELGLEDCV<br>NEIDAQQVFGYALFKDGKSTRLSYPLKEFDSDVTGKSF<br>HNGRFIQRMREKAATLANVKLEQGTVTSLIERKGTVK<br>GVHYKTKTGQDMTAYSPLTVVCDGCFSNLRRSLCNPK<br>VEIPSVFVALILKNCQLPYPKHGHVILANPSPILLYRISS<br>TEIRCLVDVPGKKIPSIGNGDMAHYLKTLVAPQIPFELY<br>DAFITAIEEGNMKTMANRSMPADPYPTPGALLLGDAF<br>NMRHPLTGGGMTVALSDIVVLRDLLRPLGDLNDAPAL<br>CRYLESFYTLRKPMASTINTLAGALYKVFCASPDQAR<br>NEMRQACFDYLSLGGIFSNGPVALLSGLNPRPLSLVLH<br>FFSVAIYGVGRLLLPIPSPQRAWLGARLISVSMFRSY
	Pg_S3767.15	MDFNMIVQHMLVGAIVSLLGFAVLYLLGKKNKSNSSA<br>LVQTQGKECAEWQPEISENPDIIIVGAGVAGSALACTL<br>GKDGRRVLVIERDLTEPDRIVGELLQPGGYLKLVELGL<br>EDCVNEIDAQQVFGYALFKDGKSTRLSYPLKEFDSDV<br>TGKSFHNGRFIQRMREKAATLANVKLEQGTVTSLIER<br>KGTVKGVHYKTKTGQDMTAYSPLTVVCDGCFSNLRR<br>SLCNPKVEIPSVFVALILKNCQLPYPNHGHVILANPSPIL<br>LYRISSTEIRCLVDVPGKKIPSIGNGDMAHYLKTLVAPQ<br>IPFELYDAFITAIEEGNMKTMANRSMPADPYPTPGALL<br>LGDAFNMRHPLTGGGMTVALSDIVVLRDLLRPLGDLN<br>DAPALCRYLESFYTLRKPVASTINLLAGALYKVFCASP<br>DQARNEMRQACFDYLSLGGIFSNGPVALLSGLNPRPLS<br>LVLHFFSVAIYGVGRLLLPIPSPQRAW

Species	Gene ID	Protein sequence
		LGARLISDASSIIFPIIRAEGIRQMFFPRVIPTYYRCPPSK
	Pg_S1672.1	MTSYQFTLGGAIVSFLGFAMFYLLCRNKKSDNSTNLVQIKRNECVKTAHDGECQPEIVGNPDIIIVGAGVAGSALAYTLGKDGRRVLVVERDLTEPDRIVGELLQPGGYLKLIELGLEDCVNDIDAQQVFGYALFKDGKSTRLSYPLKEFHTDVTGKSFHNGRFIQRMREKAARISNVKLEQGTVTSLIERKGTVKGVHYKTKTGQDMTAYAPLTIVCDGCFSNLRRSLCNPKVDIPSVFVALILKNCQLPYPNHGHVILANPSPILLYRISTTEIRCLVDVPGKKVPSIGNGEMTHYLKTLVAPQIPLELYDAFITTIEEGNMKTMANRSMPADPYLTPGALLLGDAFNMRHPLTGGGMTVALSDIVVLRDLLRPLGDLNDSPALCRYLESFYTLRKPVASTINTLAGALYKVFCSSPDQARNEMRQACFDYLSIGGIFSNGPVALLSGLNPRPLSLVLHFFSVAIYGVGRLLLPFPSPKRAWLGARLITDAASIIFPIVKAEGVRQMFFPIAIPAYHRSPPIN
	Pg_S4651.2	MNVLNPLKMVNLGLKLLNPDVIIVGAGVAGSALACTLGKDGRRVLVIERDLTEQDRIVGELLQPGGYLKLMELGLEDCVDEIDAQEVFGYALFKDGKSTRLSYPLKDFHSDVAGRNFHNGRFIQRMREKAATLSNVRLEQGTVTSLIEKKGTVKGVHYRTKTGQELTTCAPLTIVCDGCFSNLRRSLCNPKVEIPSHFVAMILKNCQLPYPNHGHVILANPSPILLYRVSSTEIRCLVDIPGQKVPSIGNGDMSHYLKTVVAPQIPLDLNKAFISTIEEGNIRTMANRSMPANPYPTPGALLLGDAFNMRHPLTGGGMTVALSDIVVLRDLLRPLLDFNDAPSLSKYLEAFYNLRKPVASTRNTLTGAIYKVFCASPNQARNEMRQACFDYLSLGGIFSNGPIALLSGLKPQPLSLILHFFSVAIYGVGRLLLPLPSPKRAWLGARLISDASAIIFPIIKAEGIRQMFFPSTISAYHRSPPTY
	Pg_S6081.2	MAIFMDINMIDESIFVGVFLGLVVLLLLRKRKNSHNNLVPIKIQRNECAKPPQDGESRPEISENPDVIIVGAGVAGSALACTLGKDGRRVLVIERDLTEQDRIIGELLQPGGYLKLMELGLEDCVDKIDAQEVFGYALFKDGKSTRLSYPLKDFHSDVAGRSFHNGSFIQPMREKAATLSNVRLEQGTVTSLIEKKGTVKGVHYRTKTGQELTACAPLTIVCDGCFSNLRRSLCNPKVEIPSHFVAMILKNCQLPYPNHGHVILANPSPILLYRISSTEIRCLVDIPGQKVPSIGNGDMSYYLKTVVAPQIPLDLNKAFLSAIEEGNIRTMANRSMPADPYPTPGALLLGDAFNMRQTLTGGGMTVALSDIVVLRDLLRPLRDFNDAPSLSKYLEAFYTLRKPVASTINTLAGALYKVFCASPNQARNEMRQACFDYLSLRGIFSNGPIALLSGLNPQPLSLVLHFFSVAIYGVGRLLLPLPSPKHAWLGARLISDASGIIFPIIKAEGIRQMFFPSTISAHHRSPPTY
	Pg_S0967.30	MREKAATLSNVHLEQGTVSSLLEENGTIKGVQYKTKT

Species	Gene ID	Protein sequence
		VQDVKAYAPLTIVCDGCFSNLRRSLCKPKVDVPSCFVG<br>LILENCKLPHPNHGHVILADPSPILFYPISSTEVRCLVDV<br>PGQKLPSLANGDLANYLKTMVAPQIPPELHDAFIAAID<br>KGNIRTMPNRSMPAAPYPTPGALLMGDAFNMRHSLTG<br>GGMTVALSDIVVLRNLLKPLGDMNDADSLCKYL
	Pg_S3064.7	MAISMDINMINESIFVGVLLVLVVLLLLGKRKNSHNNL<br>VPIKIQRNECAKPPQDGESRPENPDVIIVGAGVAGSALT<br>CTLGKQVEIPSHFVAMILKNCQLPYPNHGHVILANPSPI<br>LLYRISSTEIRCLVDIPGQKVPSIGNGDMSYYLKTVVAP<br>QIPLDLNKAFISAIEEGNIRTMANRSMPADPYPTPGALL<br>LGDAFNMRHPLTGGGMTVALSDIVVLRDLLRPLRDFN<br>DAPSLSKYLEAFYTLRKKMLEVIESKIKQLGFGISNLFD<br>ALLEGLIAEGLEHLMHWLMSML
	Pg_S7552.1	MKTMTNRSMPDDPYPTPGALLLGDAFNMRHPLTGEG<br>MTVALSDIAVLRDLLRSLGALNDSPALCRYLESFYTLR<br>KPVASTINTLAGALYKVFCASPDQARNEMRQACFDYL<br>SLGGIFSNGPVALLSGLNPRPLSLVLHFFSVAMYGIGRL<br>LLPIPSPKRAWLGARLISVSMRCSY
	Pg_S0341.4	MHRLVQNFVANLLLQSAVLKFNLRRKAKMSKGTGGA<br>AGAKGKKKGATFVINCGKPVEDKIMDIASLENFLQERI<br>KIGGRAGASGDSVSVVREKSKITPVASTINTLAGALYK<br>VFCASPDQARKEMREACFDYLSLGGVCSTGPVALLSG<br>LNPRPLSLVLHFFAVAIYGVGRMLLPFPSPK<br>RLWAGVRLISAASGIIFPIIQAEGVRQMFFPATVPAYYY<br>RAPPADDIKF
	Pg_S4043.9	MSKGTGGAAGAKGKKKGVTFVINCGKSVEDKIMDIA<br>SLEKFPQERIKVGGKAGALGDSVS<br>IPVASTINTLAGALYKVFCASPDQARKEMREACFDYLS<br>LGGVCSTGPVALLSGLNPRPLSLVLHFFAVAIYGVGRM<br>LLPFPSPKRLWAGVRLISAASGIIFPIIQAEGVRQMFFPA<br>TVPAYYY
	Pg_S4043.1	MSKGIGGAVGAKGKKKGATFVINCRKPVEDKIMDIAS<br>LEKFPHERIKVGGKAGALGDSVSVPVASTINTLAGALY<br>KVICASPDQARKEMREACFDYLSLGGVCSTGPVALLS<br>GLNPRPLALLMMRKKMKLFNCVSLDYAIKNHLR
	Pg_S7775.2	MTLQQILANAVGDDVILNDSNVVNFEDDGNKVTVILE<br>NGQCYEGDLLVGADGIWSKVRENLFGHKEPTYSGYT<br>CYTGIANFVPADIDTVGYRVFLGHKQYFVSSDVGGGK<br>MQWYAFHNEPAGGTDGNNDKKERLLQIFEGWCDNVI<br>DLLLETNEDAILRRDIYDQEPTFTWGKGHVTLLGDSV<br>HAMQPNLGQGGCMAIEDSYQLAMELDRACKQSNESG<br>NPTDIASSLRSYESARKVRVSVIHGLARMAAIMASIYK<br>AYLGVGLGPLSFLTKFRIPHPGTVGGRFFIDIGMPLMLS<br>WVLG

Species	Gene ID	Protein sequence
		GYGSNLEGRSPQCRLSDKANNQLKRWFEDDDALERA LSGEWFLFPVGNESSPSESIFLSRDAGNPCIIGSVPHANI LGISIVISSPQVSKLHAQISSKDGGAFFLTDLRSKHGTWI TDNEGRRYRVPPNFPARFHPSDVIEFGSDKKAAFRVKV MKIPLKTTEKKDWSEEALQAVLDLPT
Polygala tenuifolia	Pt9G00922	MLDPHTFAWIFGSVICFVVLYYLVLPYKNQRVSFEHAS NYLDNSITTTAGECRSSESKAKDVDVIIVGAGVAGAAL AHTLGKDGRRVHVIERELSEPDRIVGELLQPGGYLKLV ELGLQDCVEDIDAQRVFGYALFKDGKDTRLAYPLEKF HADVSGRSFHNGRFIQRMREKATSLPSVRLEQGTVTSL LEEKGAIRGVRYKNKDGLELTAYAPLTIVCDGCFSNLR RSLCNPKVDVPSCFVGLVLENCNLPYANHGHVILADPS PILFYPISSTEIRCLVDVPGQKVPSISNGEMEKYLKTVV APQIPPELYDAFVAAVDNGNIRTMPNRSMPAAPYPTPG ALLLGDAFNMRHPLTGGGMTVALSDIVVLRDLLRPLR DLHDAPILCKYLESFYTLRKPVASTINTLAGALYKVFC ASPDQARKEMRQACFDYLSLGGVFSKGPVSLLSGLNP RPLSLVLHFFAVAIYGVGRLLLPFPSPKRIWIGAKLISGA SGIIFPIIKAEGVRQMFLPATVPAYYRAPPIIKS
	Pt14G01074	MEVETELFAALLASLIGFVLLFILPRARAQTNARMTKA DNMNKGRQVKQCIKSLILDNCDASGTPDVIIVGSGVA GSALAYTLGKDGRRVHVIERDLTEPDRIVGELLQPGGY LKLIELGLEDCVEKIDAQQVLGYALFKDGKHTKVSYP LEKFHSDVSGRSFHNGRFIMRMRAKAASLPNVQLEEG TVTSLLEENGIVNGVHYRTKDGHEHKVYAPLTIVCDG CFSNLRRALCHPKVEVPSHFVGLVLENCQLPFANHGH VILGNPSPVLFYPISSTEIRCLVDVPGQRLPSISNGEMA NYLKTVVAPQVPSELHDAFVSAIDKGQIRTMPNRTMP ADPHPTPGALLMGDAFNMRHPLTGGGMTVALSDIAVL RDLLKPLHDFNDSPALCKYLESFYTLRKPVASTINTLA GALYKVFCASPDEARTEMRQACFDYLSLGGVFSTGPV ALLSGLNPRPLSLVLHFFAVAIYGVGRLMLPFPSPKRM WIGIRLVLSASGIIFPIIKGEGVRQMFFPATIPAYYQSSLL R
	Pt1G02478	MLDPHTFAWIFGSVICFVALYYLVLPFKNQRVSFEHAS NYLDNSITTTAGECRSSESKAKDVDIIIVGAGVAGAAL AHTLGKDGRRVHVIERDLSEPDRIVGELLQPGGYLKLV ELGLQDCVEDIDAQRVFGYALFKDGKDIRLAYPLEKFH ADVSGRSFHNGRFIQRMREKATSLLSVRLEQGTVTSLL EEKGAIRGVRYKNKDGLELTAYAPLTIVCDGCFSNLRR SLCNPKVDVPSCFVGLVLENCNLPYANHGHVILADPSP ILFYPISSTEIRCLVDVPGQKVPSISNGEMEKYLKTVVA PQIPPELYDAFVAAVDNGNIRTMPNKSMPAAPYPTPGA

Species	Gene ID	Protein sequence
		LLLGDAFNMRHPLTGGGMTVALSDIVVLRDLLSPLRD LHDAPTLCRYLESFYTLRKPVASTINTLAGALYKVFCA SPDQARKEMCQACFDYLSLGGFFSKGPVSLLSGLNPR PLSLVLHFFAVAIYGVGRLLLPFPSPKRIWIGARLISGAS GIIFPIIKAEGVRQMFLPAIVPSFYRAPSIEVINKK
	Pt13G01952	MFSLKPSFLEPLQMEYQYLLAAIVSSTMLIIFILYSLEA KRKMRTSSFIYHNACHMGSVNVVEKEVDGETDIIVVG AGVAGSALAYTLGKDGRRVQVIERDLSEPDRIVGELLQ PGGYLKLIELGLEDCVNEIDAQRIYGYALYKDGKSTRT PYPLENFPPDVAGRSFHNGRFIQRMRGKAASLPNVRLE QGTVTSLLEENGTVKGVSYKTKSGQELLATAPLTIVCD GCFSNLRRSLCNPKVEIPSCFVGLVLENCNLPHANHGH VILADPSPILFYPISSNEIRCLVDVPGQKVPSVGNGEMA RYLKSVVAPQIPPELYDAFIAAIDKGNIRTMPNRSMPAS PHPTPGALLMGDAFNMRHPLTGGGMTVALSDIVLLRN LLRPLRNLNDSTSLCKYLESFYTLRKPMASTINTLAGA LYKVFCASPDPARKEMRDACFDYLSLGGVFTNGPTSL LSGLNSRPSSLVVHFFAVAVYGVGRLLIPFPTPRRLWIG ARLISGATAIIFPIIKAEGVRQMFFPVTVPAYYRAPPVHL HA
	Pt18G00164	MGSVDGMLEKTVDRETDIIVIGAGVAGSALAYTLGKD GRRVHVIERDLSEPDRIVGELLQPGGYLKLIELGLEDC VNEIDAQRIYGYALYKDGRSTRTPYPLKNLPSDVAGRS FHNGRFIQRMREKAASLPNVRLEQGTVISLLEENGTVK GVSYKNKNGQELIATAPLTIVCDGCFSNLRRSLCNPKV ESPSCFVGLLLESCDLPHANHGHVILADPSPILFYPISSN EIRCLVDVPGQKVPSVGNGEMARYLKIVVAPQVPSELY DAFIAAIDKGNIRTMPNRSMPASPYPTPGALLMGDAFN MRHPLTGGGMTVALSDIVLLRNLLRPLRNLNDSTALC KYLESFYTLRKPVASTINTLAGALYKVFCASPDPARKE MRDACFDYLSLGGVFTNGPTSLLSGLNSRPSTLVIHFFA VAVYGVGRLLIPFPTPQRLWIGARLISGATAIIFPIIKAEG VRQMFFPVTVPAYYRAPPIHLHA
	Pt12G00280	MEVDTELCIVATTLLISLFGFVLLFSVPRARAHANTRTT ETGNINKYQQVQCLKSLSSDISDSPGTSDVIVVGAGVA GSALAYTLGKKDGRRVHVIERDLTEPDRIVGELLHPGG YLKLLELGLEDCLETIDAQRVLGFALFKDGKHSRLAYP LEKLHSDVSGRSFHNGRFIMRMREKAASLPNLQLEQG TVTSLLEENGIIKGVHYKTKDGQERKVYAPLTIVCDGC FSNLRRALCHPKVEVASHFVGLVLENCGLPFANHGHVI LGNPSLVLFYPISSTEIRCMVDVPGEKLPSIANGEMAN YLKNVVAPQVPSELHDALVSAIDKGKFRTMPNRIMPA DPHPTPGALLIGDALNMRHPLTGGGMTVALSDIVVLR

Species	Gene ID	Protein sequence
		DLLKPLRDLNDVPALCKYLESFYTLRKPIASIINTMAG<br>VVYKVSCASSDESRTEIRQVCFDYLSLGGVFSTGPAAL<br>LSGLNPRPLSLVLHSFAVAIYGVGRLMLPFPSPKRIWKG<br>VRFILSASGIIFPIIQGEGVRQMLVPATTQAYYKSLTNEM<br>TALTKIIVGAAAGIKESFNRLSTASKRFSLKSSSVVVS
	Pt2G00562	MRQKAATLSNVRLEQGTVTSLIEENGTVKGVSYKTKN<br>GQLLTATASLTVVCDGCFSNLRRTLCKPKVEIPSSFVG<br>MILENCNLPHANHGHVILGDPSPILFYPISSNEIRCLVD<br>VPGHKVPSIANGEMASYLKTAVAPQIPAELHDAFLAAI<br>EKGSIRTMTNRSMPASPCLTPGALLLGDAFNMRHPLTG<br>GMTVALFRGLLRPLDDLSDSVSLCEYLDSFYTLRKPVA<br>ATINTLAGALYNVFCTPPDPARNELRQACFDYLSLGGV<br>FSSGPIALLSGLNPQPMNLVLHFFSVAIYGIGRLLVPFPS<br>PLRMWIGVRLILDASAIIFPIIKAEGLRQMFFPVTVPAFL<br>KDPYVQNVPYSLVLKNNMENHLFNVQNL
	Pt2G00563	MEYHYLLAAMVAFTMVIMFVLYSSEARKQEKGCLSV<br>CGNGCQKTSDNGLGETEIDSRTDIIIVGAGVAGSALAY<br>TLGKDGRRVHVIERDLSEPDRIVGELLQPGGYLKLVEL<br>GLEDCVNEIDAQHVYGYALYKGGRSTRISYPLENFNT<br>DVAGRSFHNGRFVQRMRQKAATLSNVRLEQGTVTSLI<br>EENGTVKGVSYKTKNGQLLTATAPLTVVCDGCFSNLR<br>HTLCKPKVEIPSSFIGMILENCNLPHANHGHVILGDPSP<br>ILFYPISSNEIRCLVDVPGQKVPSIANGEMASYLKTAV<br>APQIPAELHDAFLAAIEKGSIRTTTNRSMPASPCPTPGA<br>LLLGDAFNMRHPLTGGGMTVALSDIVLLRDLLRPLDD<br>LSDSVSLCEYLDSFYTLRKVCIRPNRTTNVIIWESLDT
	Pt18G00371	MERFRLAYCCLQRILLLAGTGTSSCLGKNRWSTAAYA<br>LEATAVTRHTVIQIYPMMRIFLFFVLFLCYSCRSLAAIE<br>KGNIRTMTNRSMSASPCPTSCALLLGDACNMRRPLTG<br>GGMTVALSDIVLLRDLLKNCMYSQLRPGRPRKIQPPIV<br>PFAPATVQDITPPNDVERTVQVALAKASMVDDLQNKN<br>QELMKQIEICQEENKILDKMHRQKVAEVEKLTQTIREL<br>EEAVLAGGAAANAVRDYRRKFQEMNEERKTLDRELA<br>RAKVTANRVAVVVANEWKDAMSATEYY
Arabidopsis thaliana.	AT1G58440.1	MESQLWNWILPLLISSLLISFVAFYGFFVKPKRNGLRH<br>DRKTVSTVTSDVGSVNITGDTVADVIVVGAGVAGSAL<br>AYTLGKDKRRVHVIERDLSEPDRIVGELLQPGGYLKLL<br>ELGIEDCVEEIDAQRVYGYALFKNGKRIRLAYPLEKFH<br>EDVSGRSFHNGRFIQRMREKAASLPNVQLEQGTVLSL<br>LEENGTIKGVRYKNKAGEEQTAFAALTIVCDGCFSNLR<br>RSLCNPQVEVPSCFVGLVLENCNLPYANHGHVVLADP<br>SPILMYPISSTEVRCLVDVPGQKVPSIANGEMKNYLK<br>TVVAPQMPHEVYDSFIAAVDKGNIKSMPNRSMPASPY

Species	Gene ID	Protein sequence
		PTPGALLMGDAFNMRHPLTGGGMTVALADIVVLRNL<br>LRPLRDLSDGASLCKYLESFYTLRKPVAATINTLANAL<br>YQVFCSSENEARNEMREACFDYLGLGGMCTSGPVSLL<br>SGLNPRPLTLVCHFFAVAVYGVIRLLIPFPSPKRIWLGAK<br>LISGASGIIFPIIKAEGVRQMFFPATVPAYYYKAPTVGET<br>KCS
	AT2G22830.1	MKPFVIRNLPRFQSTLRSSLLYTNHRPSSRFSLSTRRFT<br>TGATYIRRWKATAAQTLKLSAVNSTVMMKPAKIALDQ<br>FIASLFTFLLLYILRRSSNKNKKNRGLVVSQNDTVSKNL<br>ETEVDSGTDVIIVGAGVAGSALAHTLGKEGRRVHVIER<br>DFSEQDRIVGELLQPGGYLKLIELGLEDCVKKIDAQRV<br>LGYVLFKDGKHTKLAYPLETFDSDVAGRSFHNGRFVQ<br>RMREKALTLSNVRLEQGTVTSLLEEHGTIKGVRYRTK<br>EGNEFRSFAPLTIVCDGCFSNLRRSLCKPKVDVPSTFVG<br>LVLENCELPFANHGHVVLGDPSPILMYPISSSEVRCLVD<br>VPGQKLPPIANGEMAKYLKTRVAPQVPTKVREAFITAV<br>EKGNIRTMPNRSMPADPIPTPGALLLGDAFNMRHPLTG<br>GGMTVALADIVVLRDLLRPIRNLNDKEALSKYIESFYT<br>LRKPVASTINTLADALYKVFLASSDEARTEMREACFDY<br>LSLGGVFSSGPVALLSGLNPRPLSLVLHFFAVAIYAVCR<br>LMLPFPSIESFWLGARIISSASSIIFPIIKAEGVRQMFFPR<br>TIPAIYRAPP
	AT4G37760.1	MAPTIFVDHCILTTTFVASLFAFLLLYVLRRRSKTIHGS<br>VNVRNGTLTVKSGTDVDIIIVGAGVAGAALAHTLGKE<br>GRRVHVIERDLTEPDRIVGELLQPGGYLKLIELGLEDC<br>VKDIDAQRVLGYALFKDGKHTKLSYPLDQFDSDVAGR<br>SFHNGRFVQRMREKASLLPNVRMEQGTVTSLVEENGII<br>KGVQYKTKDGQELKSFAPLTIVCDGCFSNLRRSLCKPK<br>VEVPSNFVGLVLENCELPFPNHGHVVLGDPSPILFYPIS<br>SSEVRCLVDVPGSKLPSVASGEMAHHLKTMVAPQVPP<br>QIRDAFISAVEKGNIRTMPNRSMPADPIHTPGALLLGDA<br>FNMRHPLTGGGMTVALSDIVILRDLLNPLVDLTNKESL<br>SKYIESFYTLRKPVASTINTLAGALYKVFLASPDDARSE<br>MRRACFDYLSLGGVCSSGPVALLSGLNPRPMSLVLHFF<br>AVAIFGVGRLLVPLPSVKRLWLGARLISSASGIIFPIIKAE<br>GVRQMFFPRTIPAIYRAPPTPSSSSPQ
	AT5G24150.1	MAFTNVCLWTLLAFMLTWTVFYVTNRGKKATQLADA<br>VVEEREDGATDVIIVGAGVGGSALAYALAKDGRRVHV<br>IERDLREPERIMGEFMQPGGRLMLSKLGLEDCLEGIDA<br>QKATGMTVYKDGKEAVASFPVDNNNFPFDPSARSFHN<br>GRFVQRLRQKASSLPNVRLEEGTVKSLIEEKGVIKGVT<br>YKNSAGEETTALAPLTVVCDGCYSNLRRSLNDNNAEV<br>LSYQVGFISKNCQLEEPEKLKLIMSKPSFTMLYQISSTD

Species	Gene ID	Protein sequence
		VRCVFEVLPNNIPSISNGEMATFVKNTIAPQVPLKLRK<br>IFLKGIDEGEHIKAMPTKKMTATLSEKKGVILLGDAFN<br>MRHPAIASGMMVLLSDILILRRLLQPLSNLGNAQKISQ<br>VIKSFYDIRKPMSATVNTLGNAFSQVLVASTDEAKEAM<br>RQGCYDYLSSGGFRTSGMMALLGGMNPRPISLIYHLC<br>AITLSSIGHLLSPFPSPLRIWHSLRLFGLAMKMLVPHLK<br>AEGVSQMLFPVNAAAYSKSYMAATAL
	AT5G24160.1	MAFTHVCLWTLVAFVLTWTVFYLTNMKKKATDLADT<br>VAEDQKDGAADVIIVGAGVGGSALAYALAKDGRRVH<br>VIERDMREPERMMGEFMQPGGRLMLSKLGLQDCLED<br>IDAQKATGLAVYKDGKEADAPFPVDNNNFSYEPSARS<br>FHNGRFVQQLRRKAFSLSNVRLEEGTVKSLLEEKGVV<br>KGVTYKNKEGEETTALAPLTVVCDGCYSNLRRSLNDD<br>NNAEIMSYIVGYISKNCRLEEPEKLHLILSKPSFTMVYQ<br>ISSTDVRCGFEVLPENFPSIANGEMSTFMKNTIVPQVPP<br>KLRKIFLKGIDEGAHIKVVPAKRMTSTLSKKKGVIVLG<br>DAFNMRHPVVASGMMVLLSDILILRRLLQPLSNLGDA<br>NKVSEVINSFYDIRKPMSATVNTLGNAFSQVLIGSTDE<br>AKEAMRQGVYDYLCSGGFRTSGMMALLGGMNPRPL<br>SLVYHLCAITLSSIGQLLSPFPSPLRIWHSLKLFGLAMK<br>MLVPNLKAEGVSQMLFPANAAAYHKSYMAATTL
	AT5G24140.1	MTYAWLWTLLAFVLTWMVFHLIKMKKAATGDLEAEA<br>EARRDGATDVIIVGAGVAGASLAYALAKDGRRVHVIE<br>RDLKEPQRFMGELMQAGGRFMLAQLGLEDCLEDIDA<br>QEAKSLAIYKDGKHATLPFPDDKSFPHEPVGRLLRNGR<br>LVQRLRQKAASLSNVQLEEGTVKSLIEEEGVVKGVTY<br>KNSAGEEITAFAPLTVVCDGCYSNLRRSLVDNTEEVLS<br>YMVGYVTKNSRLEDPHSLHLIFSKPLVCVIYQITSDEV<br>RCVAEVPADSIPSISNGEMSTFLKKSMAPQIPETGNLRE<br>IFLKGIEEGLPEIKSTATKSMSSRLCDKRGVIVLGDAFN<br>MRHPIIASGMMVALSDICILRNLLKPLPNLSNTKKVSDL<br>VKSFYIIRKPMSATVNTLASIFSQVLVATTDEAREGMRQ<br>GCFNYLARGDFKTRGLMTILGGMNPHPLTLVLHLVAIT<br>LTSMGHLLSPFPSPRRFWHSLRILAWALQMLGAHLVDE<br>GFKEMLIPTNAAAYRRNYIATTTV
Oryza sativa	LOC_Os03g12910.<br>2	MKTGGRSPPLLPPSPPLPINRNNLEASARLRLLLPPPPP<br>LLPFSPLARRRLAATRIGAAPRPQQRSPPPRAERPRRPH<br>HYWCGMAEVAAGTGQLIGVAVATLLAAIFLAAALLGS<br>RRRRRRAPLAGKPAAVGGCGVADGEGCGGDGRTDVI<br>VVGAGVAGSALAYTLGKDGRRVHVIERDLTEPDRIVG<br>ELLQPGGYLKLIELGLEDCVQEIDAQRVLGYALFKDGK<br>DTKLSYPLEKFHSDVAGRSFHNGRFIQRMRQKAASLP<br>NVQLEQGTVTSLVEEDGTVKGVKYKTKSGEELKAYAP

Species	Gene ID	Protein sequence
		LTIVCDGCFSNLRRALCSPKVDVPSCFVGLVLENCQLP<br>HANHGHVVLANPSPILFYPISSTEVRCLVDVPGQKVPSI<br>ANGEMAKYLKTVVAPQIPPEIYDSFIAAIDKGSIRTMPN<br>RSMPAAPHPTPGALLMGDAFNMRHPLTGGGMTVALS<br>DIVVLRNLLKPLRNLHDASALCKYLESFYTLRKPVAST<br>INTLAGALYKVFSASPDQARNEMRQACFDYLSLGGVF<br>SNGPIALLSGLNPRPLSLVAHFFAVAIYGVGRLMLPLPS<br>PKRMWIGVRLISSFHCRVHVV
	LOC_Os03g12900.<br>5	MTEPDRIVGELLQPGGYLKLMELGLEDCVEEIDAQRV<br>LGYALLKDGRNTKLSYPLEKFHSDVAGRSFHNGRFIQ<br>KMRQKAASLPNVHLEQGTVTSLLEEGGTVKGVQYKT<br>KSGEELKAYAPLTIVCDGCFSNLRRVLCSPKVDVPSCF<br>VGLVLENCQLPHPNHGHVILANPSPILCYPISSTEIRCLV<br>DIPGQKVPSMATGEMAKYLKTVVAPQIPPELHDSFIAA<br>IDKGSIRTMPNRSMPAAPLPTPGALLMGDAFNMRHPLT<br>GGGMTVAFSDIVVLRNLLKPLGNLHDAPSLCKYLESF<br>YTLRKPVASTINTLAGALYKVFCASTDQAKNEMREAC<br>FDYLSLGGVFSNGPIALLSGLNPRPLSLVAHFFAVAIYG<br>VGRLMLPVPSPKRMWIGARLVSGACGIIFPIIKAEGVR<br>QMFFPATVPAYYRAPPPMESG
	LOC_Os03g12900.<br>1	MDAVAGAGQLVGLAAATLLTAAFLVAVKMGWRRRRR<br>QREVAPEGGCRVVGGDGGDRTDIVIVGAGVAGSALAY<br>TLGKDGRRVHVIERDMTEPDRIVGELLQPGGYLKLME<br>LGLEDCVEEIDAQRVLGYALLKDGRNTKLSYPLEKFHS<br>DVAGRSFHNGRFIQKMRQKAASLPNVHLEQGTVTSLL<br>EEGGTVKGVQYKTKSGEELKAYAPLTIVCDGCFSNLRR<br>VLCSPKVDVPSCFVGLVLENCQLPHPNHGHVILANPSP<br>ILCYPISSTEIRCLVDIPGQKVPSMATGEMAKYLKTVVA<br>PQIPPELHDSFIAAIDKGSIRTMPNRSMPAAPLPTPGALL<br>MGDAFNMRHPLTGGGMTVAFSDIVVLRNLLKPLGNL<br>HDAPSLCKYLESFYTLRKPVASTINTLAGALYKVFCAS<br>TDQAKNEMREACFDYLSLGGVFSNGPIALLSGLNPRP<br>LSLVAHFFAVAIYGVGRLMLPVPSPKRMWIGARLVSGA<br>CGIIFPIIKAEGVRQMFFPATVPAYYRAPPPMDQIRITHI<br>NAILHRSCRLCPLISELLEAEKGTEVPCLFRHRTLPFKR<br>LNACTGDMTYTYMQ
Platycodon grandifloru m	PgSE6	MIMELLSMKMVEEFMLEVVIATLFGFAVFYFLGSEKKS<br>AERALIKTMDKRTNECVKTSGEDAQCSRGDTDQGKT<br>DIIIVGAGVAGSALACSLGKDGRRVLVIERDLTLQDRIV<br>DCVNDIDAQRVFGYALFKDGKSTRLSYPLEKFHSDVS<br>GRSFHNGRFIQRMREKAATISNVTLEQGTVTSLIEEKG<br>TIKGVQYKTKTGQEMTASAPLTIVCDGCFSNLRRGLC<br>KPEVDIPSCFVALILKNCDVPFPNHGHVFLADPSPILCY

Species	Gene ID	Protein sequence
		PISSTEIRLLVDIPGQKVPSVSNGEMTHYLKTVVASQIPP<br>ELHSAFIAAIEEGNIRTMANRSMPAAPQPTPGALLLGD<br>AFNMRHPLTGGGMTVALSDIVILRDLLRPLDNLDDPSA<br>LCNYLELFYTLRKPVASTINTLAGALYKVFCASPDEAR<br>NELRQACFDYLSLGGVFSIGPVSLLSGLNPKPMSLVFH<br>FFSVAIFGVGRLLLPFPSLKRAWLGARLIADASSIIFPIIR<br>AEGAGRMFFPAAVPAYYRSPFTH
	PgSE2	MDKLMQTQGRKIFRQKLQQYASDGEISEEEVKALERL<br>QNCDLPYKNHGHVILADPSPILFYQISDTEVRCLVDVA<br>GEWIPPELYTAFLAAIDKGNIRTMPNRSMPADPQPTLG<br>ALLLGDAFNMRHPLTGGGMTVALSDIVILRDLFRPIRD<br>LNDASTLANYLESFYTLRKPVASTINTLAGALPAVSVFP<br>ATSTTISGFRLLLFPAASTIVFE
	PgSE3	MSCLRTMPNRSMPADPQPTPGALLLGDAFNMRHPLTG<br>GGMTVALSDIVILRDLFRPIRDLNDASTLANYLESFYTL<br>RKVRLMAIKLYVSFYTLRKVGDFGLARVLPTLDRYNL<br>SSKVQSALGYMAPESNFAVVYLFCCYVVYLYIAYIR
	PgSE1	MLALNPPTSLLLLDPSFPLHYSQFKIRSQLHRPRERNRS<br>ISATFNKTRSESEKNQVSISSLSSSLTVTQFPVKKKMLT<br>VDGTLIVATLFASVFGFVFLHILGRLNNSNKVKPAYSEI<br>RCDPSTARECGPNNGSEIDVVIVGAGVAGAALAHTLG<br>KEGRRVQVIERDLTEPDRIVGELLQPGGYLKLIELGLE<br>DCVEEIDAQRVLGYALFKDGRNTRVSYPLEKFHTDVS<br>GRSFHNGRFIQKMREKAATLPNVRLEQGTVTSLIEENG<br>TVKGVQYKTKNGQELKAYAPLTIVCDGCFSNLRRSLC<br>NPKVEVPSCFVGLILENCKLPYPNHGHVILADPSPILLY<br>PISSTEVRCLVDVPGQKLPSLANGEMAKYLKTNVAPQ<br>VPPELHDAFVAAIEKGNIRTMPNRSMPADPHPTPGALL<br>MGDAFNMRHPLTGGGMTVALSDICVLRDLLKSLHDM<br>RDADSLCKYLESFYTLRKPVASTINTLAGALYKVFCAS<br>SDQARTEMREACFDYLSLGGVFSTGPVALLSGLNPRPL<br>SLVLHFFAVAVYGVGRLLVPFPSPKRLWIGARLISNASG<br>IIFPIIKAEGIRQMFFPASVPAYYRAPRPSDSR
	PgSE4	MADQYLLGLILASLLGSLLLYRLIVKKNARECSMEVST<br>TATTATTTTSINGECRSRNDADIIIVGAGVAGAALAHTL<br>GMEERRVHVIERDLTEPDRIVGELLQPGGYLKLIELGL<br>EDCVEEIDAQRVFGYALFKNGKSTRLSYPLEKFHKDVS<br>GRSFHNGRFIQRMREKSSLLPNVRLEQGTVTSLLEENG<br>IIKGVQYKSKTGEEMKAYAPLTIVCDGCFSNLRRSLCN<br>PKVDVPSCFVGLVLENCQLPHENHGHVILADPSPILFY<br>RISSTEIRCLVDVPGQRVPSISNGDLSKYLKTVVAPQIPP<br>ELYDAFIATVDKGNIRTMPNRSMPAAPHPTPGALLMG<br>DAFNMRHPLTGGGMTVALSDIVVLRNLLRPLHDLNDA

Species	Gene ID	Protein sequence
		PTLCKYLESFYTLRKPVASTINTLAGALYKVFCASPDQARKEMREACFDYLSLGGVFSTGPVSLLSGLNPRPMSLVLHFFAVAIYGVGRLLLPFPSPKRMWIGARLITSASGIIFPIIKAEGVRQMFFPATVAAYYRAPPAK
	PgSE7	MANSSSSTTSSPSMHAYAMVELYTMGNLQYLVAGTIAFLLGFVVLYNSTRRSRNIFNGGLAEVGELDSGVKRSGNGQSFRSDTAGNTDVIIVGAGVAGAALACTLAKDGRQVHVIERDLTEPDRIVGELLQPGGYQKLIELGLEDCLNGIEAQEVFGYALYMDGKSTRLSYPLEKYHTDVSGRSFHNGRFIQRMREKAASLPNVKLEQGTVTSLLQKEGTVHGVQYKTKDGKEMTAYAPLTIVCDGCFSNLRRSLCKPKVEVPSCFVGLVLENCDLPYKNHGHVILADPSPILFYQISDTEVRCLVDVPGEKVPSISNGEMGRYLKSVVAPQIPPELYTAFLAAIDKGNIRTMPNRSMPADPQPTPGALLLGDAFNMRHPLTGGGMTVALSDIVILRDLFRPIRDLNDASTLANYLESFYTLRKVRLVAIKLYVSCFYDVFEKRTKKEQKKKVELSSIYGGFPVASTINTLAGALYRVFCASPDPARKEMRDACFDYLSLGGIFTDGPTALLSGLNPRPLSLFLHFFAVAIYGVGRLLIPFPSAKRLWLGIRLISGASGIIFPIIKSEGVRQMFFPGTVPAYYRGPPVG
	PgSE5	MAEQLILAVVIAVLLGFAVLYLLGKSKKKGKIATKFSSQTNESVNITKEVSQSLPETAGNVDIIVVGAGVAGSALACAIGKDGRQVLVIERDLALQDRIVGELLQPGGYLKLIELGLEGKQDVYCVRNIDAQNVYGYALFKDGKSTKLTYPLEKFDSDVCGRSFHNGRFIQRMREKAATIANVRLEQGTVTSLLEEKGTIKGVQYKTKAGQEMTACAPLTIVCDGCFSNLRRGLCKPEVDIPSCFVALLLKNCEVPYPNHGHVFLANPSPILCYRISSTEIRLLVDIPGQKVPSISNGEMAHYLKTMVAPQIPPELHGAFMTAIEEGNIRTMANRSMPAAPQPTPGAILLGDAFNMRHPLTGGGMTVALSDIVVLLNLLRPLKNLNDASALCKYLDSFYTLRKPVASTINTLAGALYKVFSASPDQARIELRQACFDYLSLGGVFSTGPVSLLSGLNPKPMTLVLHFFSVAIFGVGRLLLPYPSPKRTWLGARLISDASSIIFPIIRAEGAGQMFFSTAVPAYYSSPFTH
